# Tick-borne pathogens and body condition of cattle in smallholder rural livestock production systems in East and West Africa

**DOI:** 10.1186/s13071-023-05709-0

**Published:** 2023-03-30

**Authors:** Dieter J. A. Heylen, Bersissa Kumsa, Elikira Kimbita, Mwiine Nobert Frank, Dennis Muhanguzi, Frans Jongejan, Safiou Bienvenu Adehan, Alassane Toure, Fred Aboagye-Antwi, Ndudim Isaac Ogo, Nick Juleff, Dionne Crafford, Josephus Fourie, Michel Labuchange, Maxime Madder

**Affiliations:** 1 Evolutionary Ecology Group, Department of Biology, University of Antwerp, Wilrijk, Belgium; 2 Eco-Epidemiology Group, Department of Biomedical Sciences, Institute of Tropical Medicine, Antwerp, Belgium; 3 Interuniversity Institute for Biostatistics and Statistical Bioinformatics, Hasselt University, Diepenbeek, Belgium; 4 Department of Parasitology, College of Veterinary Medicine and Agriculture, Addis Ababa University, Bishoftu, Ethiopia; 5 Department of Veterinary Microbiology and Parasitology, College of Veterinary Medicine and Biomedical Sciences, Sokoine University of Agriculture, PO Box 3019, Morogoro, Tanzania; 6 Department of Bio-Molecular Resources and Bio-Laboratory Sciences (BBS), College of Veterinary Medicine, Makerere University, Kampala, Uganda; 7 Department of Veterinary Tropical Diseases, Faculty of Veterinary Science, University of Pretoria, Onderstepoort, South Africa; 8 Zootechnical, Veterinary and Halieutic Research Laboratory (LRZVH), National Institute of Agricultural Research (INRAB), 01 BP 884, Cotonou, Benin; 9 Université Nangui Abrogoua, UFR Sciences de La Nature, 02 Bp 801, Abidjan 02, Côte d’Ivoire; 10 Department of Animal Biology and Conservation Science, School of Biological Sciences, College of Basic and Applied Sciences, University of Ghana, Legon-Accra, Ghana; 11 National Veterinary Research Institute, Vom, Plateau State Nigeria; 12 Bill & Melinda Gates Foundation, Seattle, WA USA; 13 Clinvet International Pty (Ltd), 1479 Talmadge Hill South, Waverly, NY 14892 USA; 14 Clinomics, Bloemfontein, South Africa; 15 Clinglobal, B03/04, The Tamarin Commercial Hub, Jacaranda Avenue, Tamarin, 90903 Mauritius

**Keywords:** *Anaplasma marginale*, *Anaplasma centrale*, *Babesia bigemina*, *Babesia bovis*, *Ehrlichia ruminantium*, *Theileria parva*, Vector competence, Sub-Sahara Africa

## Abstract

**Background:**

The majority of the African population lives in rural areas where they heavily depend on crop and livestock production for their livelihoods. Given their socio-economic importance, we initiated a standardized multi-country (Benin, Burkina Faso, Ghana, Nigeria, Ethiopia Tanzania and Uganda) surveillance study to assess the current status of important tick-borne haemoparasites (TBHPs) of cattle.

**Methods:**

We assessed pathogen prevalences (*Anaplasma marginale, Anaplasma centrale, Babesia bigemina, Babesia bovis, Ehrlichia ruminantium*, and *Theileria parva*) in the blood of 6447 animals spread over fourteen districts (two districts per country). In addition, we screened for intrinsic (sex, weight, body condition) and extrinsic (husbandry, tick exposure) risk factors as predictors of infections with TBHPs.

**Results:**

There was a large macro-geographic variation observed in *A. marginale, B. bigemina, B. bovis* and *E. ruminantium* prevalences. Most correlated with the co-occurrence of their specific sets of vector-competent ticks. Highest numbers of infected cattle were found in Ghana and Benin, and lowest in Burkina Faso. While *T. parva* was seldomly found (Uganda only: 3.0%), *A. marginale* was found in each country with a prevalence of at least 40%. *Babesia bovis* infected individuals had lower body condition scores. Age (as estimated via body weight) was higher in *A. marginale* infected cattle, but was negatively correlated with *B. bigemina* and *E. ruminantium* prevalences. *Ehrlichia ruminantium* infection was more often found in males, and *A. marginale* more often in transhumance farming. High levels of co-infection, especially the combination *A. marginale* × *B. bigemina*, were observed in all countries, except for Uganda and Burkina Faso. *Babesia bigemina* was more or less often observed than expected by chance, when cattle were also co-infected with *E. ruminantium* or *A. marginale*, respectively.

**Conclusions:**

Tick-borne pathogens of cattle are ubiquitous in African’s smallholder cattle production systems. Our standardized study will help a wide range of stakeholders to provide recommendations for TBHP surveillance and prevention in cattle, especially for *B. bovis* which heavily impacts production and continues its spread over the African continent via the invasive *Rhipicephalus microplus* tick.

**Graphical Abstract:**

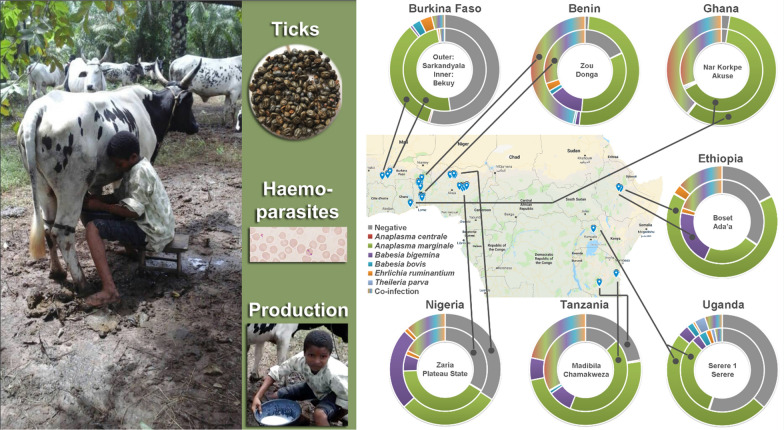

**Supplementary Information:**

The online version contains supplementary material available at 10.1186/s13071-023-05709-0.

## Background

Most of the African populations live in rural areas where they heavily depend on crop and livestock production for their livelihoods [1]. A large proportion of sub-Saharan farmers belong to resource-constrained farming communities and struggle to maintain minimal life standards, not seldomly because of the presence of livestock vector-borne and infectious diseases. To increase livestock productivity, profitability, and sustainability, affordable yield-enhancing inputs are needed. Herein, parasite control is of paramount importance, for which standardized surveys of the current state of tick-borne haemoparasites (TBHPs) are essential. In an ever-changing world, of which the anthropogenic drivers of urbanization and climate change are affecting habitat and parasite exposure risk, up-to-date surveys are key to providing African institutions (private and governmental) with opportunities to interact, collaborate and enhance mutual capacity building for parasite control. In addition, this baseline data allows for the identification and measurement of effective changes in presence and burdens of ticks and tick-borne diseases. Furthermore, because of the lack of standardized and affordable diagnostics in many African countries, it is far not possible to estimate the real burden and risk of tick-borne parasites and their associated economic damage.

As part of a parasite mapping project which focused on enhancing livestock care across Africa, we initiated a standardized multi-country surveillance study to assess the current status of important endemic TBHPs of cattle across seven sub-Saharan African territories (West Africa: Burkina Faso, Ghana, Benin, Nigeria and East Africa: Ethiopia, Uganda, Tanzania) [2, 3]. Through the creation of a sustainable veterinary network for Africa, we simultaneously sampled representative numbers of cattle across countries at the macro-geographic scale (district level) for molecular TBHP screenings, while considering the micro-geographic (farms within district) and individual (cattle within farm) variation in TBHP prevalence, and this over a time window of approximately one year. Spatio-temporal variation of following disease-causing micro-parasites of socio-economic importance [4] were investigated: (1) *Ehrlichia ruminantium*, the causal agent of heartwater, is an obligate intracellular bacterium invading endothelial cells in cattle, sheep, goats and wild ruminants with a frequently fatal outcome. Control and prevention strategies against heartwater have been developed, but with limited efficacy. *Amblyomma* ticks are considered as main vectors [5]. (2) *Anaplasma marginale*, the etiological agent of bovine anaplasmosis, is the most prevalent tick-borne pathogen of cattle, and is transmitted—often mechanically—by a broad variety of tick species. Also tabanids and stable flies are involved in its transmission cycles. It is an intraerythrocytic pathogen and causes symptoms of acute disease: anemia, weight loss, and often death. Life-long persistent *A. marginale* infection in animals that survive anaplasmosis, which are often clinically healthy, serve as reservoirs for transmission of *A. marginale* [6]. (3) A second less pathogenic species, the intraerythrocytic *A. centrale*, causes benign infections, and is often used as a vaccine strain against the more virulent *A. marginale* [6]. In subtropical regions of the world, bovine babesiosis is caused by intra-erythrocytic protozoan parasites including (4) *B. bovis* and (5) *B. bigemina* [7]. Symptoms of infection by different *Babesia* spp. are similar, but *B. bovis* is more virulent than the other piroplasms. *Babesia bigemina* is more widespread and common in Africa than *B. bovis* likely due to a wider spectrum of vector-competent *Rhipicephalus* tick species. (6) East Coast fever (*Theileria parva*) belongs to the same order as Babesidae, but shows a different type of pathology, as they can infect endothelial and white blood cells as well [8]. *Theileria parva* is transmitted mainly by *R. appendiculatus* and causes a fatal disease in susceptible animals and especially calves [4, 9, 10].

TBHP prevalence depends on a multifaceted presence and abundance of multiple susceptible hosts in suitable tick-habitat, as well as of the competent tick vectors themselves. Implementation of effective measures to control vector-borne diseases (i.e. establishment of proper treatment strategies and prevention) relies on the elucidation of these local pathogen transmission dynamics, which include the level of susceptibility and infectiousness of organisms that carry and transmit the pathogens (i.e. reservoir hosts and vector-competent ectoparasites) and contact-rates between (infected) vectors and hosts [11–13]. Here, to inspire current and future intervention plans, aside from an updated cross-sectional TBHP surveillance, we explored associations between the animal’s infection status and intrinsic characteristics as well as co-occurrences of ticks and TBHPs. In summary, in this article the following questions are addressed: (1) To which extent do TBHPs vary macro-geographically?, (2) Which host characteristics (sex, condition, and body weight as a proxy for age) are associated with TBHP prevalence?, (3) After taking into account the contribution of known vector-competent ticks, are there other vector-competent tick suspects that explain additional variation in pathogen presence?, (4) Do certain pathogen combinations occur more often than randomly expected by chance at the individual level?

## Methods

### Study site and design

**Fig. 1:**
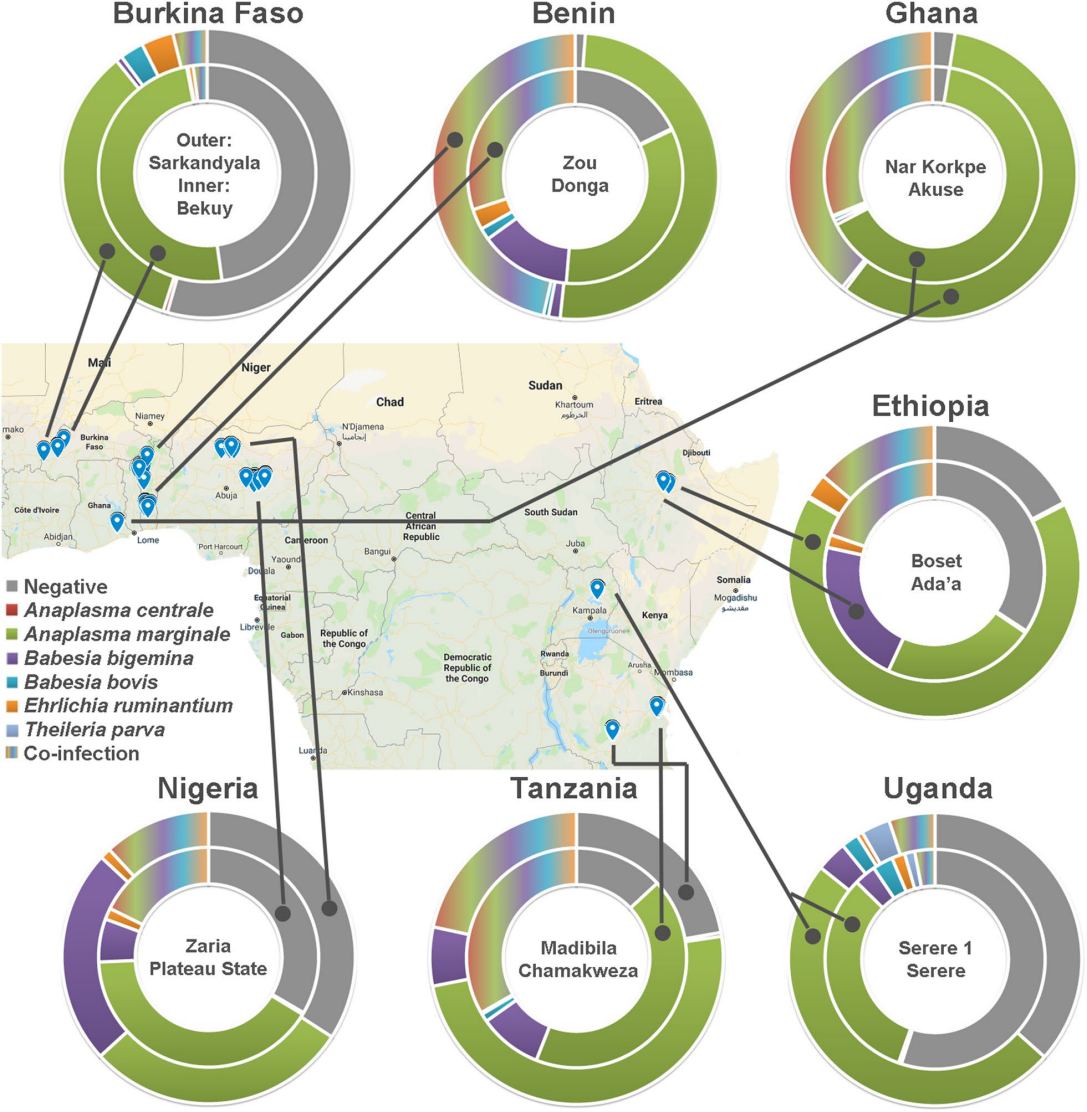
Overview of the sampling locations in seven African countries. For each of the districts, prevalence of single- and co-infected individuals with TBHP’s (see legend) are depicted in donut charts (see Additional file 1: Table S2 for more detailed distribution of co-infections within cattle individuals)

The seven countries included in the study were limited to those prioritized in Sub-Saharan Africa by the Bill & Melinda Gates Foundation's original agricultural development strategy, and were considered to give a good representation of cattle TBHP’s of East and West Africa. In each country, two districts (Fig. 1) were selected with known high cattle density, hence with expected high prevalence of ticks and tick-borne diseases. Two sites sampled are localized in the South West Burkina Faso: Sarkandiala in the province of La Léraba and Bekuy in the province of Houet. Ghana: Akuse and Narh Korkpe are both located in the Lower Manya Krobo district. Both communities are located around the southern banks of the Volta Lake. Benin: Djougou, N’dali, OuassaPehunco, Bassila and Ouaké belong to the Donga Department; Abomey, Djidja, Ouinhi, Zangnannando and Zogbodomey, belong to the Zou Department. The Manya Krobo district is approximately 80–85 km east of Accra, the capital of Ghana. This district falls within the coastal savannah zone of the Accra plains, with a bimodal rainfall pattern. Nigeria: Plateau State (Quanpam, Shandam, and Wase) and Kaduna state (Zaria). Ethiopia: typical representative sites in central and eastern Oromia; Ada'a is located in the central Ethiopian highland at approximately 2300 m, whereas Boset is situated in the lowlands of Ethiopia at an altitude of 947 m above sea level. Uganda: Serere district, bordered by Soroti and Kaberamaido Districts to the north, Ngora District to the east, Pallisa, Kaliro, and Buyende districts the south. The district is made up of two rural counties (Kasilo and Serere), and eight sub-counties (Bugondo, Kadungulu, Pingire, Labor, Atiira, Kateta, Chere and Serere/Olio) containing 254 cattle owning villages. Tanzania: Chamakweza in Bagamoyo district (Coast region) and Madibila (Mbarali district; Mbeya region lying at an altitude of 1560 m above sea level). Sampling sites consisted of smallholder livestock farmer (resource constrained cattle breeders) settlements with mainly sedentary cattle herds located in the selected localities. The survey was conducted over a period of one year (August 2016 to June 2017). Fieldwork consisted of four quaternary sampling visits. Individual sampling duration for each visit (to both sites) was approximately two weeks (approximately 1 week per site), but varied depending on logistical challenges encountered. During each visit, the target was to sample 240 cattle (approximately 120 per site).

### Assessment of intrinsic and extrinsic ecological risk factors

The weight of cattle (sampled for parasites from small holder herds) was estimated using a RONDO® tape (Kyron Labs, Johannesburg, South Africa) according to the manufacturer’s recommendations. Body conditioning scoring was conducted for these animals according to work instructions provided in the protocol (see Additional File 2: Protocol). In addition, we determined gender, the type of husbandry, and the number of vector-competent ticks for each of the pathogens (see further). The among farm variation for each of the countries is presented in Table 1. Blood samples were taken for molecular screening of the TBHPs.

**Table 1:** Macro-geographic variation in the continuous variables included in the Generalized Linear Mixed Models

	Burkina Faso (N° farms: 19)	Ghana (7)	Benin (19)	Nigeria (7)	Ethiopia (24)	Uganda (89)	Tanzania (10)
Vector-competent tick loads							
*Anaplasma marginale*	3.35 ± 1.77	7.03 ± 3.54	65.2 ± 95.81	5.01 ± 6.49	2.68 ± 3.64	13.46 ± 12.11	31.95 ± 31.12
*Ehrlichia ruminantium*	0.51 ± 0.40	14.91 ± 9.07	32.94 ± 30.84	2.11 ± 1.99	9.95 ± 12.45	20.91 ± 18.89	5.8 ± 4.27
*Babesia bovis*	2.17 ± 1.72	6.31 ± 3.15	65.2 ± 95.81	0.00 ± 0.00	0.00 ± 0.00	6.42 ± 8.53	24.41 ± 29.17
*Babesia bigemina*	2.17 ± 1.72	6.32 ± 3.16	65.2 ± 95.81	5.01 ± 6.49	1.86 ± 3.22	6.43 ± 8.54	27.7 ± 26.64
Body measures							
Condition female	4.83 ± 1.79	5.43 ± 0.41	6.17 ± 0.4	4.46 ± 0.36	4.74 ± 1.01	5.93 ± 1.18	5.37 ± 0.67
male	5.03 ± 1.78	5.29 ± 0.49	6.11 ± 0.41	4.39 ± 0.31	5.06 ± 0.95	5.88 ± 0.79	5.49 ± 0.78
Body weight female	182.85 ± 37.31	93.95 ± 20.04	151.9 ± 40.99	135.16 ± 17.56	122.02 ± 21.8	203.88 ± 51.03	126.03 ± 27.22
male	186.21 ± 44.31	95.32 ± 21.18	152.31 ± 42.22	121.47 ± 22.74	130.4 ± 23.53	227.64 ± 58.1	119.86 ± 22.68

Data are presented as averages at farm level ± 1 standard deviation

### Blood collection and processing

Whole blood samples were collected by laceration of the middle ear vein of each sampled animal using 22 G lancing needles. About 125 μL of the blood sample was collected from the blood drops that formed on the lacerated ear vein using heparinized capillary tubes and applied onto Classic Flinders Technology Associates [FTA] Cards (Whatman®). Blood samples were allowed to air-dry and then labeled serially. Thereafter, blood samples were packed in FTA pouches with a silica gel desiccant (Sigma Aldrich, Co., Life sciences, USA) prior to shipment to Clinvet international [Uitsig Road, Universitas, Bloemfontein, 9338, South Africa] for analysis. The Pouches were appropriately labelled so that the samples can be traced back to Village, Parish, subcounty, county, district and country of orgin. Additional data on the possible intrinsic and extrinsic predictors of infection with different TBHPs were recorded on a separate Data capture form that accompanied the samples to Clinvet international.

### DNA extraction

FTA card technology was used to ship blood samples. Cards were punched (3 × 5 mm diameter punches) and subjected to DNA isolation. DNA was isolated from 2 × 5 mm diameter punches using the MagMAX™ DNA Multi-Sample Ultra Kit according to the manufacturer’s recommendations. DNA was isolated from 92 samples per run, including extraction controls for each run using a KingFisher 96-flex instrument. DNA was eluted in a final volume of 75 µl.

### Molecular identification of TBHPs

Synthetic DNA (GeneArt, Germany) encompassing the full-length PCR target amplicons were cloned into a plasmid and served as positive control templates in each PCR run. The full-length amplicon synthetic sequences were based on sequence data obtained from GenBank (Additional File 1: Table S0). Negative control samples contained bovine genomic DNA to exclude host derived detection signals. The protocol listed below is based on primer/probes from published assays [14–17]. The inhibitor tolerant and highly processive SsoAdvanced Universal Probes Supermix (Bio-Rad) DNA polymerase master mix was used in all PCR assays. Assay primers and probes were evaluated in a single target and multiplex environment using sequence verified linear synthetic DNA templates to determine the limit of detection (LOD) for each assay in the presence of 10 ng bovine DNA. Final multiplex PCR assay combinations were based on results that exhibited the same LOD in both single and multiplex PCR setup. The Hamilton Nimbus robotic liquid handling system was used for mastermix and template addition to a 384-well PCR plate. All plates contained the relevant positive control samples (synthetic DNA representing 100 copies of each target region per reaction), negative control (10 ng bovine DNA from a Clinvet donor animal to exclude host related amplification), extraction control (to exclude extraction kit related amplification). A total of 5 µl template DNA was used in a 15 µl final PCR reaction and was subjected to thermal cycling consisting of initial denaturation at 98 °C for 3 min, followed by 45 cycles of 95 °C for 15 s and 60 °C for 30 s making use of the QuantStudio6 qPCR system. Data captured during the thermal cycling was analyzed using the QuantStudio Real-Time PCR Software v1.2. Multiplex 1 was performed first to ensure that no template derived inhibition were observed (using the IAC), then followed by the other 2 PCRs. Samples exhibiting the correct amplification profiles (shape and Ct crossing within the limit of detection range) were called as ‘*Detected*’, whereas the rest were called ‘*Not detected*’.

### Tick collection and identification

In brief, half-body sampling was performed on five predilection sites: (i) the inner and outer forelegs, hind legs and abdomen; (ii) tail and anal area; (iii) head and neck; (iv) lateral area and dorsal area from shoulders to tail base; and (v) ears. The ticks were removed using forceps. The collection was performed for about 15 min in total from all the predilection sites. For heavily infested animals, the ticks remaining on the animal after the 15-min collection period were counted and recorded. The different genera were recorded separately. Ticks were identified based on morphology using a stereoscope (80-fold magnification). For better visualizing the hypostome dentition of ticks belonging to the subgenus *Rhipicephalus* (*Boophilus*), a microscope (100 to 200-fold magnification) was used. Only adult specimens were identified to species level using both taxonomic descriptions [18] and morphological keys [19, 20]. Results on geographic variation on tick communities and loads at tick species level will be published as a separate paper (Heylen D. and Madder M. in prep.). Most important competent vectors [18] are listed in Additional file 1: Table S1, together with other commonly found tick species. Per pathogen, for each host individual the sum of the number of vector-competent ticks was calculated. The among farm variation in average loads of vector-competent ticks is presented in Table 1.

### Statistical analysis

The data possess a hierarchical structure with infection levels (0/1) for each animal nested within farm, and farm nested within district. In order to obtain valid statistical inferences, the dependence structure of the data needed to be taken into account. For these purposes, Generalized Linear Mixed Models (GLMM’s) were fitted onto the data [see 21] taking into account the statistical dependence of observations within farms (nested within districts) by adding random effects at each of the levels. The residuals for pathogen proportions were assumed to follow a binomial distribution (logit-link). For the sampled cattle (*n* = 7072), the pathogen occurrence was included as response variables in models, with the following explanatory variables: the individual’s intrinsic (sex, body condition, body weight) and extrinsic risk factors (husbandry: communal vs. transhumance). In addition, we included for each individual the load of vector-competent ticks. For each of the continuous explanatory variable (i.e. tick load, body weight and body condition score) we mean-centred the data at farm, and district level, because of substantial geographic variation (Table 1). Doing so, outcomes allowed to differentiate at which level (i.e. individual, farm, district level) and how the variation in pathogen prevalence was explained. Our emphasis will lie on associations found at lower levels (i.e. individual and farm) as these are less confounded by ecological biases. We consider generalized continent-wise comparison among the four visits to be of little epidemiological relevance, give that for each country climatic seasonality differs. Therefore, the investigation of differences between visits was restricted within district, by adding the temporal macro-geographic variation as nested random effect within district.

For all analyses, a stepwise backward selection procedure was used to select the best model. At each step we excluded the fixed factor with the highest non-significant *P*-value (*P* > 0.05), re-ran the model and examined the P-values of the fixed factors in the reduced model. Model reduction continued until only significant factors (*P* < 0.05) and their lower order interaction terms were left [22].

In a second phase, residuals from each model (i.e. residuals that remain after correction of intrinsic, extrinsic risk factors as well as the known vector-competent ticks) were used to answer following questions: (1) which of the TBHPs do non-randomly co-occur at the individual level. The R package HMSC (Hierarchical Model of Species Communities) [23] was used to explore potential associations, taking into account the nested random effect structure. For this, a probit model to presence/absence data ensured successful convergence, of which posterior distributions were sampled (three Markov chain Monte Carlo chains, each with 1500 samples, thin 1000 and transient 750,000). Associations were additionally verified by adding one of the pathogens as explanatory for the other one in the GLMM’s described above. (2) which other ticks can be suspected as additional vector-competent species for a certain pathogen. For the statistical analysis of the latter, we put forward following two criteria: (a) the suspected tick species shows limited correlation with known competent vectors, otherwise geographical/ecological co-occurrence would result in potential associations with the TBHPs (Spearman rank ρ between the competent and suspected tick < 0.3); (b) it shows a positive association with the TBHPs, after the correction of the effects of the known vector-competent ticks; (c) on average 0.5 ticks are found on the animal (at the level of districts) meaning the tick is sufficiently common. After applying these selection criteria, only for the two *Babesia* species additional ticks could be evaluated for vector-competence (*Hyalomma rufipes*, *Rhipicephalus pulchellus*, *Rhipicephalus appendiculatus*, *Rhipicephalus evertsi evertsi*). For *Anaplasma* and *Ehrlichia ruminantium*, all potential vector candidates correlated with the true vector-competent ticks (ρ’s > 0.3). All data management and statistical analyses were done in SAS v 9.3 (SAS Institute, Cary, North Carolina, USA).

## Results

### Macro-geographic variation in tick-borne pathogen prevalences

**Fig. 2:**
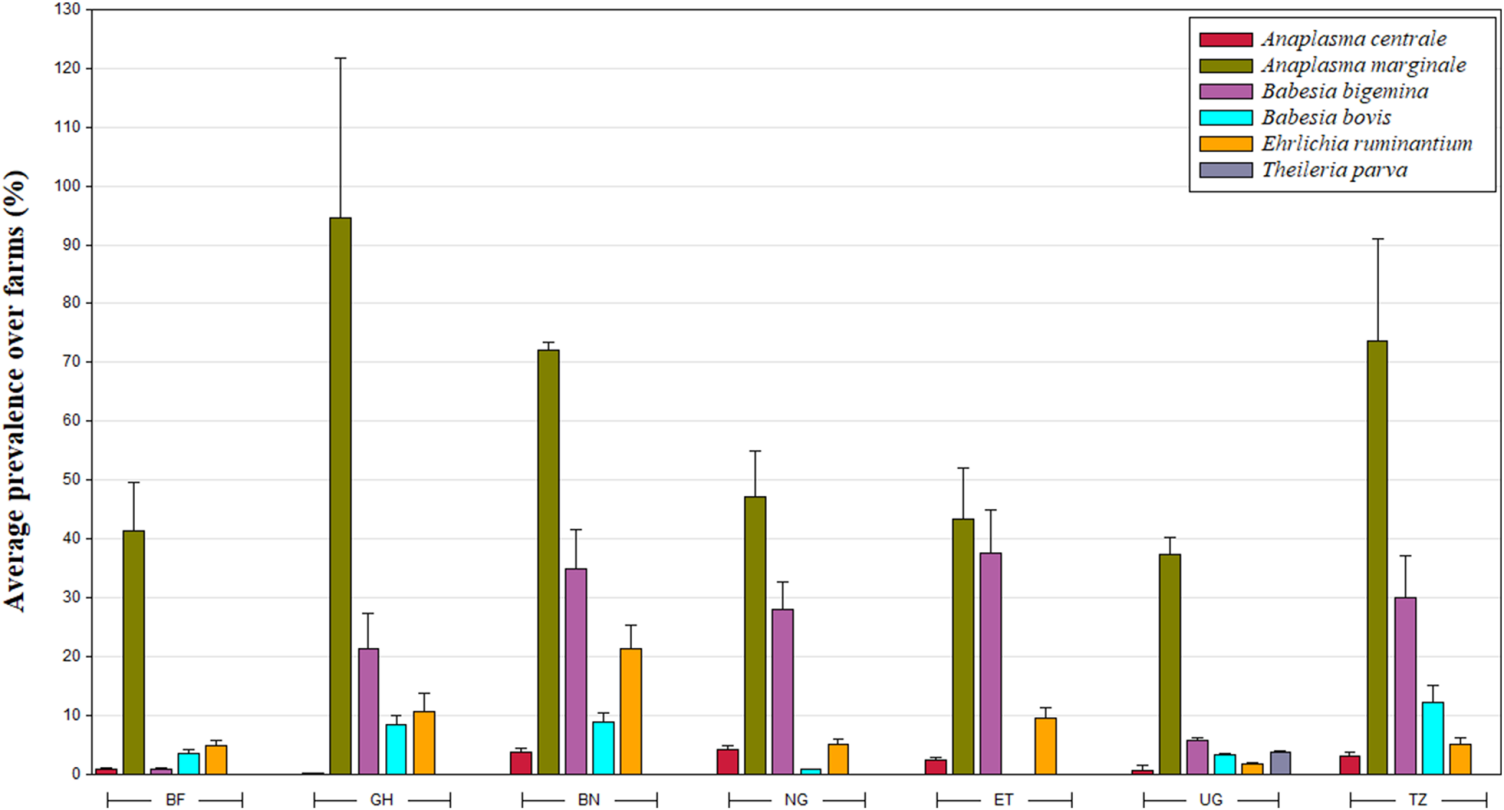
Macro-geographic variation in pathogen prevalence. Overall averages (+ 1 standard deviation) are calculated over the different farms (nested within district). *BF* Burkina Faso, *GH* Ghana, *BN* Benin, *NG* Nigeria, *ET* Ethiopia, *UG* Uganda, *TZ* Tanzania

Countries differed from each other when considering the animal’s TBHP prevalence, and for several TBHP-country combinations also significant seasonal variation was observed (see Table 2 for pairwise-comparisons between countries and visits, and Fig. 2 for macro-geographic overview). *Anaplasma marginale* was by far the most prevalent pathogen, with Ghana having the highest prevalence (92.1 to 100%). The congeneric *A. centrale* was less prevalent (< 5%) throughout the continent. *Ehrlichia ruminantium* prevalence varied between 3.4% (Burkina Faso) and 18% (Benin). *Babesia bigemina* was observed in relatively high numbers (> 10%), except for Uganda (< 10%) and Burkina Faso (1.0%). In contrast, the congeneric *B. bovis* was found in less than 10% of the cattle for all of the countries, and was absent in Ethiopia.

**Table 2:** Spatio-temporal variation in pathogen prevelance in the blood from cattle

Visit	1	2	Burkina Faso (%)			Ghana (%)			Benin (%)			Nigeria (%)			Ethiopia (%)			Uganda (%)			Tanzania (%)		
	3	4																					
*Anaplasma* *centrale*			0.5 a	0.8 a	X	0.0	0.0		4.6	4.7		2.5	6.25		4.2	5.0		2.1	0.0		3.3 ab	1.3 a	X
			0.5 ab	0.0 b		0.4	0.0		0.9	4.8		3.6	6.9		3.9	3.8		0.0	0.8		0.4 a	9.7 b	
*Overall*			0.5 B			0.1 B			3.9 A			4.5 A			4.2 A			0.8 B			3.7 A		
*Anaplasma* *marginale*			41.0 a	46.3ac	X	92.1	96.7		65.0 a	66.8 a	X	44.6 ab	53.3 a	X	63.3 a	66.3 a	X	40.4	41.2		70.6	67.4	
			53.9 c	23.5 b		100	97.1		95.4 c	86.9 d		50 ab	36.6 b		58.6 a	46.4 b		51.6	43.8		72.1	77.3	
*Overall*			45.3 C			96.46 E			78.62 D			47.61 C			58.7 A			44.12 C			71.9 B		
*Babesia* *bigemina*			0.9 a	0.0 a	X	18.3 a	32.2 b	X	37.9	41.3		35.8 a	23.3 b	X	20 ab	17.9 ab	X	8.8 a	1.3 b	X	37.2 a	33.5 a	X
			1.3 b	1.7 a		17.6 a	14.6 a		17.1	31.6		21.1 b	4.9 c		12.0 a	27.2 b		3.1 ab	7.5 a		21.3 b	12.2 c	
*Overall*			1.0 C			20.7 A			32.3 D			24.26 A			19.3 A			5.2 B			25.3 A		
*Babesia* *bovis*			0.5 a	2.9 a	X	15.0 a	12.4 c	X	15.8 a	4.3 b	X	1.6 a	0.4 a	X	0.0	0.0		8.8 a	0.9 b	X	13.3 a	10.5 a	X
			2.9 a	0.0 b		4.2 b	4.6 b		1.9 b	6.9 b		0 b	0 b		0.0	0.0		0.0 b	3.8 ab		2.1 b	8.0 a	
*Overall*			2.0 AB			9.1 C			7.33 C			0.65 A			0.0 A			3.4 AB			8.1 C		
*Ehrlichia* *ruminantium*			4.7 a	3.3 a	X	11.3 ab	5.4 a	X	15.4 a	27.2 b	X	2.9 a	7.9 a	X	10.4 a	3.8 b	X	2.5	1.3		9.4 a	5.4 ab	X
			3.7 ab	0.0 b		13.8 b	11.3 ab		11.6 a	17.9 ab		6.7 a	0.0 b		10.3 a	18.4 a		3.1	2.1		5.0 ab	0.8 b	
*Overall*			3.4 B			10.4 A			18.1 C			5.0 B			10.7 A			2.2 B			4.9 B		
*Theileria* *parva*			0.0	0.0		0.0	0.0		0.0	0.0		0.0	0.0		0.0	0.0		2.9	3.4		0.0	0.0	
			0.0	0.0		0.0	0.0		0.0	0.0		0.0	0.0		0.0	0.0		3.1	2.5		0.0	0.0	
*Overall*			0 A			0.0 A			0.0 A			0.0 A			0.0 A			3.0 A			0.0 A		
N° *screened*			944			960			982			775			953			936			897		

Farms in seven African countries (two districts each) were visited four times within a time window of 12 months

Shared letters indicate no difference, either among seasons (a,b,c,d; pair-wise comparisons made for each pathogen-country combination separately) or among countries (A,B,C,D; pair-wise comparisons made after correction for seasonal differences)

‘X’: seasonal differences detected

### Ecological correlations

Part of the TBHP prevalence variation was explained by the individual contrasts in infestation loads of vector-competent ticks (individual level Logit’s range: 1.48–3.72 10^–3^; all *P*’s < 0.041; Table 3), except for *B. bigemina*. For *A. marginale* prevalence, also average local infestation loads (i.e. at the farm level) showed a positive correlation (Logit: 5.73 ± 2.38 10^–3^; *t*_(247.8)_ = 2.41; P = 0.017). Cattle in transhuman herds tended to have a higher *A. marginale* prevalence than communal cattle (Logit_lrans—communal_: 0.83 ± 0.21; *t*_(1256)_ = 4.06; *P* < 0.0001). Female cattle tended to show lower *Ehrlichia* prevalences than males (Logit_female- male_: − 0.20 ± 0.09; *t*_(5872)_ = − 2.27; *P* = 0.024).

**Table 3:**
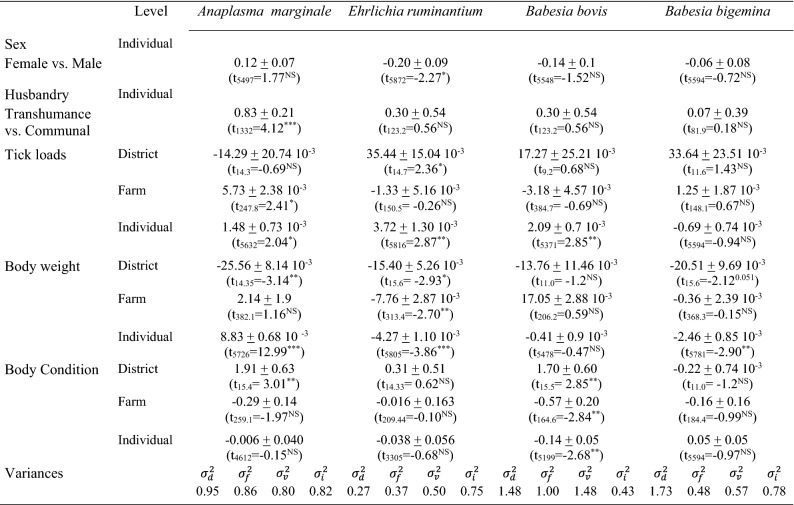
Ecological models for pathogen prevalence in cattle

Note: Parameter estimates (± standard error) from GLMM’s describing the micro-parasite prevalences (individual levels: 0, 1) in cows at the continent-wide scale (See ‘Statistical Analysis’ for details) in response to intrinsic and extrinsic risk factors. *Theileria parva* (Uganda only), *Anaplasma centrale* have been observed in low numbers, not allowing for model convergence and/or statistical testing. Tick loads of vector-competent ticks (aggregates, see Table 1) at the moment of blood sampling

${\sigma }_{d}^{2}$: variance districts; ${\sigma }_{f}^{2}$: variance between farms (nested within district); ${\sigma }_{v}^{2}$: variance visits (nested within district); ${\sigma }_{i}^{2}$: variance within farm (nested within district). Model estimates reflect the probability that ectoparasite has level ‘1’ (logit-link)

P < 0.001: ***; < 0.01: **; P < 0.05: *

**Fig. 3:**
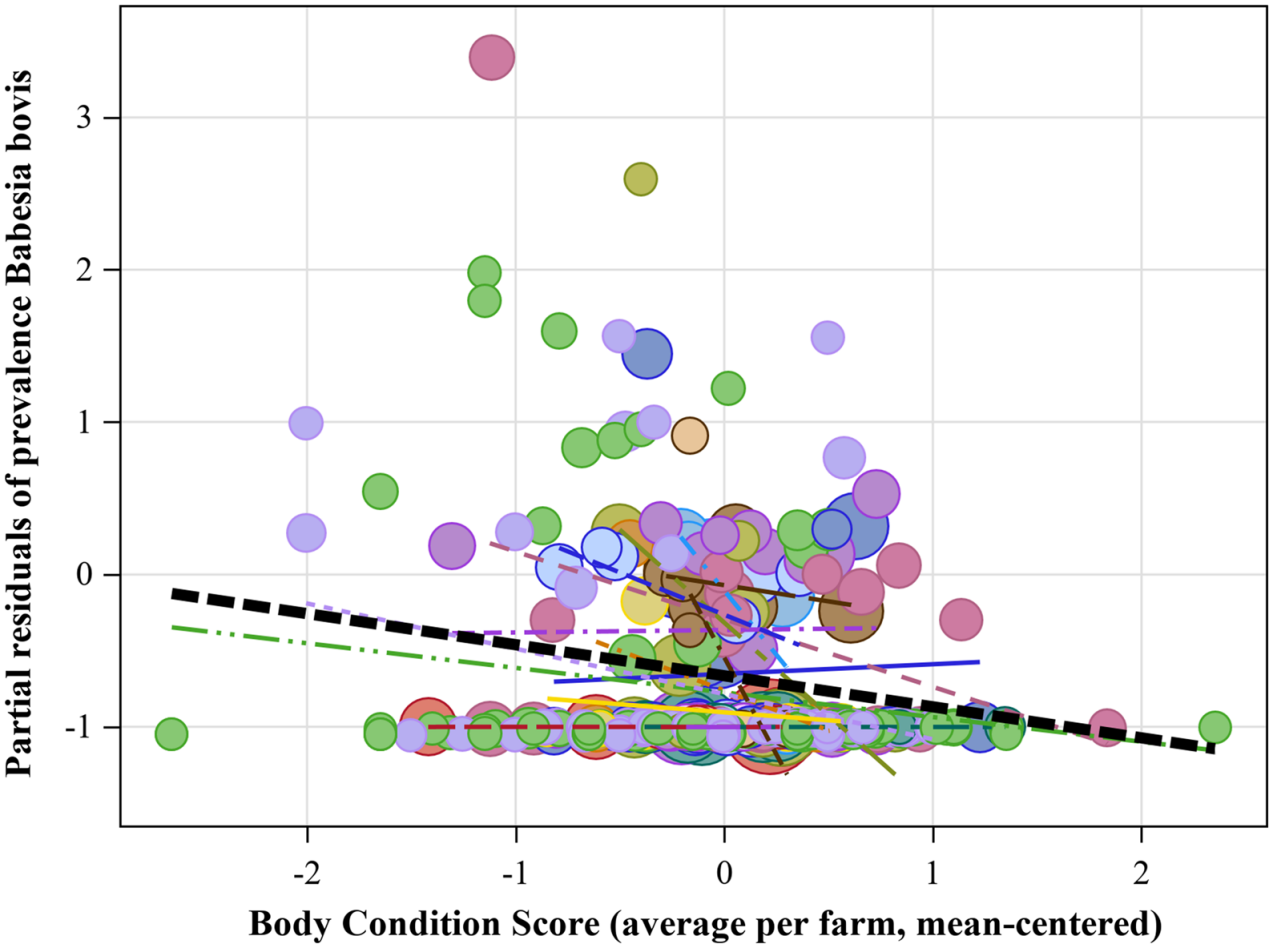
*Babesia bovis* prevalences’ partial pearson residuals (obtained from Generalized Linear Mixed Model) plotted against the body condition (average per farm, mean-centered within district). Bubble size corresponds to the number of cows sampled in each farm. For illustrative purposes, linear curves (a*x) have been added based on least squares approximation. Colours correspond to one of the 14 districts

For several pathogens, effects of body weight (a proxy for the age of the animal) were observed, though in contrasting directions: while prevalences of *A. marginale*’s showed a positive association (individual level Logit: 8.83 ± 0.68 10^–3^/kg;  *t*_(5726)_ = 12.99; *P* < 0.001), both *B. bigemina* (individual level Logit: − 2.46 ± 0.85 10^–3^/kg; *t*_(5781)_ = − 2.90; *P* = 0.0038) and *E. ruminantium* (individual level Logit: − 4.27 ± 1.1 10^–3^/kg; *t*_(5805)_ = − 3.86; *P* = 0.0001; farm level logit: − 7.76 ± 2.87 10^–3^/kg; *t*_(313.4)_ = − 2.70; *P* = 0.0073) showed negative associations. Furthermore, *Babesia bovis* prevalences were negatively correlated with body condition (a proxy for production effect) both at individual (logit: − 0.14 ± 0.05; *t*_(5199)_ = − 2.61; *P* = 0.009) and local level (logit: − 0.57 ± 0.20; *t*_(164.6)_ = − 2.82; *P* = 0.005) which is also shown by the partial residuals in Fig. 3.

### Suspected vector-competent ticks

When applying the criteria set upfront (see ‘Statistical analysis’ section), only for the *Babesia* spp. the potential vector role of additional ticks could be tested. Here, the individual variation of *Hyalomma rufipes* loads were positively associated with *B. bigemina* prevalence (logit 19.13 ± 8.7 10^–3^; *P* = 0.028). Other tick species were negatively associated with TBHP prevalence (e.g. *Rhipicephalus pulchellus* at district level) or did no correlate at all.

### Co-occurrence of TBHPs at the individual level

After correcting for extrinsic and intrinsic risk factors at the individual level (Table 3), some pathogen combinations occurred more often than expected by chance. As indicated by the exploratory HMSC model (Additional File 1: Fig. S1) cattle was more often infected with *B. bigemina* when infected with *E. ruminantium* (logit_yes-no_: 0.39 ± 0.12; *t*_(5855)_ = 3.30; *P* = 0.001). In contrast, cattle were less often infected with *B. bigemina* when infected with *A. marginale* (logit_yes-no_: − 0.47 ± 0.03; *t*_(5724)_ = − 5.30; *P* < 0.0001).

## Discussion

The main objective of the study was to determine the most important cattle TBHPs in seven Sub-Saharan African countries (Table 2), and to document potential risk factors for TBHP infection. We guided the data collection via a rigorous pre-defined protocol, thus did an analysis on standardized data. Our study therefore embodies a relatively up-to-date status of cattle TBHPs in small-scale rural livestock production systems. Moreover, by respecting the hierarchical structure of the data in our analyses, we could investigate associations at the individual level—which is the least affected by ecological biases.

More than 70% of the animals sampled were infected with at least one TBHP. While several TBHPs occurred in high (*Anaplasma marginale*) to medium (*Babesia bigemina* and *Ehrlichia ruminantium*) prevalences—of which most also showed significant spatial variation—*Theileria parva* was nearly absent from all sampled sites. A possible explanation for latter observation is the high mortality caused by East Coast fever in areas where the vector *R. appendiculatus* is present (Uganda and Tanzania), especially in young and susceptible animals [4]. Only acutely infected and/or carrier animals can be found positive, but—especially for the latter group—sensitivity of diagnostic methods is often too low to detect circulating antigens.

Without having any thorough knowledge on local host abundances and diversity, several of the geographical patterns turn out to be the consequence of the tick biology, in particular their vector-competence and occurrence. Macro-geographic variation in one tick species, *Hyalomma rufipes*, coincided with the presence of *B. bovis* in the animal’s blood. Although this tick has not been considered as vector-competent for this *Babesia* species, it turns out to be for *Babesia occultans* [24]. Given that *H. rufipes* does not share the same ecological niches of *Babesia bovis*-competent *Rhipicephalus* ticks, further research on its link with the pathogen is required. Husbandry played a role in *A. marginale*, in that transhumance farmed cattle were more often infected than communal animals. This difference may be the result of higher exposure risks to ticks and flies from wild large herbivores in the former group. Of the intrinsic exposure risk factors, the animal’s gender explained part of the variation in *E. ruminantium* prevalence, in that females had significantly lower prevalences than males. Immunosuppressive effects of androids which would increase tick and/or TBHP susceptibility, could be at the physiological basis of this observation, in addition to behavioural factors like sex differences in roaming and grooming [13]. Contrasting effects with body weight—a proxy for the cattle age—were found as well: in *A. marginale* body weight was positively associated, which could be the effect of cumulative exposure risk with age and/or higher pathogen tolerance in the heavier animal. Alternatively, as bovine anaplasmosis causes reduced weight gain, and recovered animals require a long period of convalescence during which they are less productive [6], higher mortality in lighter animals have led to the observed positive association with body weight. In contrast, in the more virulent *E. ruminantium* and *B. bigemina*, body weight was negatively associated with pathogen prevalence, which could be the effect of acquired immunity (more likely to develop with age) and carrier status of the animal, higher levels of resistance in heavier animals and/or pathogen-affected survival rates. Without experiments, it is difficult to exclude alternative interpretations like age-related differences in exposure or innate resistance [13].

Highest prevalence of *B. bovis* was linked to animals with the lowest body condition score, confirming high pathogenicity levels that comes with this pathogen and/or higher susceptibility in animals of lower conditions, inevitably resulting in significant production losses. It is the first time that the effect of *B. bovis* prevalence on body condition score was evaluated using a uniform and standardised method over such a vast geographic area. Considering the ever-extending spread of *R. microplus* (the most important vector *B. bovis*) in Western [25] and Eastern Africa [2] but also in other sub-tropical and tropical areas of the globe, production losses might remain underestimated and therefore also the importance of the control of ticks. As this monotropic, one-host tick has developed resistant strains in several continents towards different classes of acaricides [26] it remains the most important acarological threat to cattle globally. The other TBHP’s did not show any association with body condition scores, which may be due to some level of endemic stability and carrier status. However, as long as *Babesia bovis*’ R0—which inevitably is linked to the biology and life history of ticks and host [27] as well as synergistic/antagonistic interactions with co-infecting parasites—is sufficiently large, the pathogen can thrive without a need to reduce virulence [28].

One of the limitations of this observational study is the differentiation between correlation and causation in the above-mentioned associations between TBHP occurrence and the cattle’s general health measures. Assessing the pathology (e.g. anemia, icterus) would help understanding better whether the PCR-positive signals are linked to more pathogen-specific harm, and thus would also result in a better assessment of local socio-economic impacts by the TBHP’s. In the absence of experimental longitudinal data—controlling for ecological stressors affecting the animal’s health—cause and consequence in observations that include health impairments are difficult to disentangle.

The co-infections found in individuals strongly suggest that cattle are permissive for multiple pathogens. The most frequent observed co-infection was *A. marginale* × *B. bigemina*, but also *A. marginale* × *E. ruminantium* was frequently observed. The occurrence of *B. bigemina* was more likely when *E. ruminantium* was present in the blood, i.e. this combination was much higher than expected from the prevalence of each pathogen separately. It could be the result of variation in general susceptibility among individual animals, but could also indicate transmission and/or proliferation facilitation between two pathogenic agents. *Babesia bigemina* was less likely found when the animal was infected with *A. marginale*. Cross-immunity is unlikely given that both pathogens have different antigen repertoires, but a higher immune readiness caused by the infection with one TBHP could lead to a lower susceptibility towards the other TBHP [29]. The pathways that lead to facilitation and/or inhibition can only be elucidated with experimental studies in which parasite-driven physiological, cellular and biochemical interactions are disentangled [30, 31].

## Conclusions

This standardized surveillance underscores the importance of tick-borne pathogens of cattle in Sub-Saharan Africa, with co-parasitism being the rule rather than the exception. Future studies could also include wildlife host surveys, tick densities in the off-host environment, detailed habitat characteristics and specific resources that may support dense populations of ticks and hence the circulation of TBHPs. Isolates of relevant parasite strains should be evaluated for the effectiveness of different pharmaceutical and biological products, which may result in more effective control strategies. As transboundary movement of cattle between African countries is a major risk factor when governing vector-borne diseases in Africa, genetic population studies of relevant strains may also provide further insights in the spread and invasion of TBHP’s. Integration of this knowledge with a good understanding of current complexities in socio-economic and climate changes will enable policymakers and scientists to provide prevention strategies.

## Supplementary Information

**Additional file 1: Table S0.** Synthetic positive controls. Table S1. Vector-competent ticks for the four tick-borne pathogens covered in the ecological analysis. **Table S2.** Distribution of (co-)infections in cattle individuals. **Fig. S1.** Heatmap of TBHPs species-to-species associations (co-infections) based on the HMSC models.

**Additional file 2:** Protocol.

## Data Availability

The datasets generated and/or analysed during the current study are not publicly available due to Contract Research Organization agreements (data will be stored in the archives of Clinglobal, Mauritius), but are available from the corresponding author on reasonable request.

## Abbreviations

DNA: Deoxyribonucleic acid
GLMM: Generalized Linear Mixed Model
qPCR: Quantitative real-time polymerase chain reaction
