# Supplementary material for: Tick-borne pathogens and body condition of cattle in smallholder rural livestock production systems in East and West Africa
Source: Parasit Vectors. 2023 Mar 30;16:117. doi: 10.1186/s13071-023-05709-0 (PMC10064580; doi:10.1186/s13071-023-05709-0)
Supplement: Supplementary file 1 — Additional file 1: Table S0. Synthetic positive controls. Table S1. Vector-competent ticks for the four tick-borne pathogens covered in the ecological analysis. Table S2. Distribution of (co-)infections in cattle individuals. Fig. S1. Heatmap of TBHPs species-to-species associations (co-infections) based on the HMSC models. [file 13071_2023_5709_MOESM1_ESM.docx]

Additional File 1

Table S0. Synthetic positive controls.

| **Target organism** | **Accession number used to generate the synthetic positive control** | **Reference** |
| --- | --- | --- |
| *Anaplasma centrale* | AF414867 | ^1^Decaro *et al*., 2008 |
| *Anaplasma marginale* | M59845 | ^1^Decaro *et al*., 2008 |
| *Babesia bigemina* | AY603402 | ^2^Kim *et al*., 2007 |
| *Babesia bovis* | AY603398 | ^2^Kim *et al*., 2007 |
| *Ehrlichia ruminantium* | AY236058 | ^3^Steyn *et al*., 2007 |
| *Theileria parva* | L02366 | ^4^Papli *et al*., 2011 |
| **References**  ^1^ Duplex real-time polymerase chain reaction for simultaneous detection and quantification of *Anaplasma marginale* and *Anaplasma centrale*. J Vet Diagn Invest. 2008;20:606-11.  ^2^ Kim C, Iseki H, Herbas MS, Yokoyama N, Suzuki H, Xuan X, et al. Development of TaqMan-based real-time PCR assays for diagnostic detection of *Babesia bovis* and *Babesia bigemina*. Am J Trop Med Hyg. 2007;77:837-41.  ^3^ Steyn HC, Pretorius A, McCrindle CME, Steinmann CML, Van Kleef M. A quantitative real-time PCR assay for *Ehrlichia ruminantium* using pCS20. Vet Microbiol. 2008;131:258-65.  ^4^ Papli N, Landt O, Fleischer C, Koekemoer JO, Mans BJ, Pienaar R, et al. Evaluation of a TaqMan real-time PCR for the detection of *Theileria parva* in buffalo and cattle. Vet Parasitol. 2011;175:356-9. | | |

Table S1. Vector-competent ticks for the four tick-borne pathogens covered in the ecological analysis.

|  | *Anaplasma marginale* | *Ehrlichia ruminantium* | *Babesia bigemina* | *Babesia*  *bovis* |
| --- | --- | --- | --- | --- |
|  |  |  |  |  |
| Tick species |  |  |  |  |
| *Amblyomma gemma* |  | X |  |  |
| *Amblyomma variegatum* |  | X |  |  |
| *Hyalomma rufipes* | X |  |  |  |
| *Rhipicephalus decoloratus* | X |  | X |  |
| *Rhipicephalus pulchellus* | X |  |  |  |
| *Rhipicephalus microplus* | X |  | X | X |
| *Rhipicephalus evertsi evertsi* | X |  |  |  |
| *Rhipicephalus annulatus* | X |  | X | X |
| *Rhipicephalus geigyi* |  |  |  |  |
| *Rhipicephalus appendiculatus* |  |  |  |  |
| *Hyalomma truncatum* |  |  |  |  |
| *Rhipicephalus lunulatus* |  |  |  |  |
| *Rhipicephalus praetextatus* |  |  |  |  |
|  |  |  |  |  |
| Note:  X: considered to be vector-competent.  ‘’: other tick species frequently collected, and tested for potential associations with pathogen prevalence (see ‘Statistical Analysis’) | | | | |

Table S2. Distribution of (co-)infections in cattle individuals.

| Co-infection | Overall | Burkina Faso | Benin | Ethiopia | Ghana | Nigeria | Tanzania | Uganda |
| --- | --- | --- | --- | --- | --- | --- | --- | --- |
| *A. marginale* | 60.77 | 86.45 | 46.76 | 59.60 | 62.93 | 52.05 | 56.39 | 74.80 |
| *A. marginale* × *B. bigemina* | 11.19 | 1.08 | 16.41 | 6.78 | 17.41 | 9.36 | 14.81 | 2.56 |
| *B. bigemina* | 8.46 | 0.86 | 7.70 | 14.55 | 0.53 | 23.59 | 9.51 | 6.10 |
| *A. marginale* × *E. ruminantium* | 4.98 | 2.37 | 8.71 | 6.36 | 7.37 | 2.34 | 2.45 | 0.79 |
| *A. marginale* × *B. bovis* | 2.98 | 1.29 | 2.57 | 0.00 | 7.05 | 0.78 | 5.03 | 1.18 |
| *A. marginale* × *A. centrale* | 2.27 | 0.65 | 2.01 | 3.67 | 0.11 | 5.46 | 3.80 | 0.79 |
| *A. marginale* × *B. bigemina* × *E. ruminantium* | 1.74 | 0.00 | 4.24 | 0.85 | 2.24 | 0.78 | 1.77 | 0.20 |
| *E. ruminantium* | 1.68 | 4.09 | 1.56 | 3.11 | 0.11 | 2.14 | 0.14 | 2.36 |
| *B. bigemina* × *E. ruminantium* | 1.28 | 0.00 | 2.68 | 3.11 | 0.00 | 1.95 | 0.54 | 0.20 |
| *B. bovis* | 1.11 | 2.37 | 1.23 | 0.00 | 0.32 | 0.00 | 0.68 | 4.53 |
| *A. marginale* × *B. bigemina* × *B. bovis* | 0.78 | 0.00 | 1.56 | 0.00 | 0.85 | 0.00 | 2.04 | 0.00 |
| *A. marginale* × *B. bovis* × *E. ruminantium* | 0.50 | 0.22 | 1.23 | 0.00 | 0.85 | 0.00 | 0.41 | 0.20 |
| *A. marginale* × *A. centrale* × *B. bigemina* | 0.48 | 0.00 | 1.23 | 0.71 | 0.00 | 0.78 | 0.41 | 0.00 |
| *T. parva* | 0.46 | 0.00 | 0.00 | 0.00 | 0.00 | 0.00 | 0.00 | 4.33 |
| *B. bigemina* × *B. bovis* | 0.27 | 0.00 | 0.22 | 0.00 | 0.11 | 0.19 | 1.09 | 0.20 |
| *A. marginale* × *A. centrale* × *E. ruminantium* | 0.23 | 0.00 | 0.11 | 0.99 | 0.00 | 0.39 | 0.00 | 0.20 |
| *A. centrale* | 0.21 | 0.43 | 0.11 | 0.28 | 0.00 | 0.19 | 0.27 | 0.39 |
| *A. marginale* × *B. bigemina* × *B. bovis* × *E. ruminantium* | 0.21 | 0.00 | 0.56 | 0.00 | 0.11 | 0.00 | 0.54 | 0.00 |
| *A. marginale* × *A. centrale* × *B. bigemina* × *E. ruminantium* | 0.08 | 0.00 | 0.45 | 0.00 | 0.00 | 0.00 | 0.00 | 0.00 |
| *B. bigemina* × *B. bovis* × *E. ruminantium* | 0.06 | 0.00 | 0.22 | 0.00 | 0.00 | 0.00 | 0.14 | 0.00 |
| *B. bovis* × *E. ruminantium* | 0.04 | 0.22 | 0.11 | 0.00 | 0.00 | 0.00 | 0.00 | 0.00 |
| *B. bigemina* × *T. parva* | 0.04 | 0.00 | 0.00 | 0.00 | 0.00 | 0.00 | 0.00 | 0.39 |
| *A. marginale* × *T. parva* | 0.04 | 0.00 | 0.00 | 0.00 | 0.00 | 0.00 | 0.00 | 0.39 |
| *A. marginale* × *A. centrale* × *B. bovis* | 0.04 | 0.00 | 0.22 | 0.00 | 0.00 | 0.00 | 0.00 | 0.00 |
| *E. ruminantium* × *T. parva* | 0.02 | 0.00 | 0.00 | 0.00 | 0.00 | 0.00 | 0.00 | 0.20 |
| *A. marginale* × *B. bovis* × *T. parva* | 0.02 | 0.00 | 0.00 | 0.00 | 0.00 | 0.00 | 0.00 | 0.20 |
| *A. marginale* × *A. centrale* × *B. bigemina* × *B. bovis* | 0.02 | 0.00 | 0.11 | 0.00 | 0.00 | 0.00 | 0.00 | 0.00 |
| Co-infected cows (%) | 27.27 | 5.83 | 42.64 | 22.47 | 36.1 | 22.03 | 33.03 | 7.5 |
| Infected individuals | 4762 | 465 | 896 | 708 | 936 | 513 | 736 | 508 |
| Note: Prevalences refer to the number of host individuals with one or more tick-borne pathogens. From top to bottom: most to least frequently observed (combinations of) infections. (Percentages sum to 100% for each). | | | | | | | | |


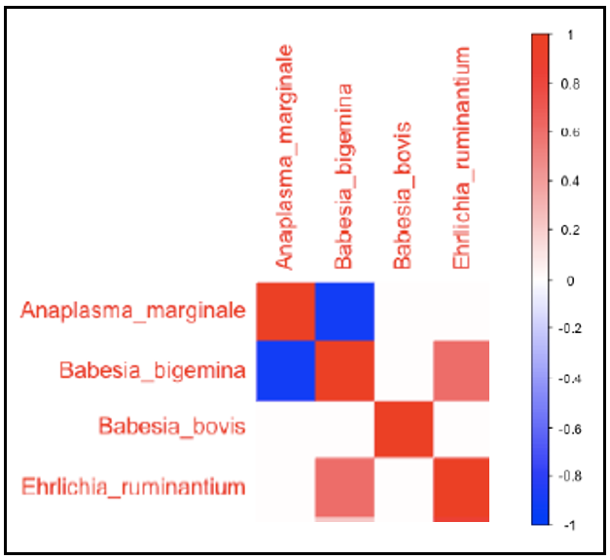


Fig. S1. Heatmap of TBHPs species-to-species associations (co-infections) based on the HMSC model with the cattle individual’s identity as a sampling unit, after controlling for covariates (including locations where the blood sample was taken). Blue and red colours show parameters that are estimated to be positive and negative, respectively, with at least 0.95 posterior probability.
