# Supplementary material for: Tick-borne pathogens and body condition of cattle in smallholder rural livestock production systems in East and West Africa
Source: Parasit Vectors. 2023 Mar 30;16:117. doi: 10.1186/s13071-023-05709-0 (PMC10064580; doi:10.1186/s13071-023-05709-0)
Supplement: Supplementary file 2 — Additional file 2: Protocol. [file 13071_2023_5709_MOESM2_ESM.docx]

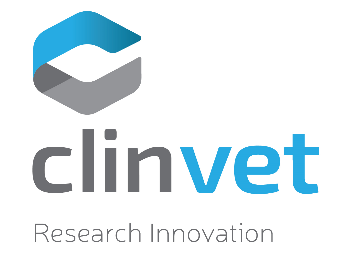


Postal: PO Box 11186
Universitas 9321 Bloemfontein

South Africa

Physical: Uitzich Road
Bainsvlei 9338 Bloemfontein

South Africa

SURVEY OF ecto- and endoparasite TAXA IMPACTING ON small-scale rural livestock farmers in BENIN

BILL AND MELINDA GATES FOUNDATION (BMGF) PROJECT

**DOCUMENT: Protocol**

**STATUS: Draft version 4**

**DATE: 27 July 2016**

PROTOCOL APPROVAL

SURVEY OF ecto- and endoparasite TAXA IMPACTING ON small-scale rural livestock farmers in BENIN

**BILL AND MELINDA GATES FOUNDATION (BMGF) PROJECT**

**QUALITY STANDARD: Non-GCP/GLP**

**Index terms:** Parasite mapping, Benin, endoparasite, ectoparasite, livestock

| **Dr SB Adehan (MSc)**  **Investigator**  **National Institute of Agricultural Research (INRAB)** |  | **Date** |
| --- | --- | --- |

| **Prof M Madder (PhD)**  **Sponsor Representative**  **Clinvet International (Pty) Ltd**  **(hereafter referred to as “Clinvet”)** |  | **Date** |
| --- | --- | --- |

**TABLE OF CONTENTS**

1 TITLE 6

1.1 Project reference 6

2 STUDY SITE AND LOCATION 6

3 STUDY CONTACTS 6

3.1 Sponsor Company 6

3.2 Sponsor Representative 6

3.3 Investigator 6

3.4 Collaborating researchers and departments of affiliation 7

4 OBJECTIVE(S) OF THE STUDY 7

5 BACKGROUND AND JUSTIFICATION FOR STUDY 8

6 SCHEDULE OF EVENTS 8

6.1 Proposed dates 8

7 STUDY DESIGN 9

7.1 Type of study and overall study design 9

7.2 Experimental unit and justification 10

7.3 Sample size and justification 10

7.3.1 Smallholder herds 10

7.3.2 Abbottoir 10

11 ASSESSMENTS 11

11.1 Weigh estimation and body condition scoring 11

11.2 Tick counts and sampling 11

11.2.1 Sampling from smallholder farms 11

11.3 Faecel flotations/sedimentations and helminth sampling 12

11.3.1 Sampling from smallholder farms 12

11.3.2 Sampling from abbotoirs 12

11.4 Blood collection and testing for haemoparasites 13

11.5 Assessment of demographic, socio-economic parameters and husbandry practices 13

11.6 Laboratory strain establishment 13

11.6.1 Tick strain colony establishment 13

11.6.2 Helminth strain colony establishment 13

12 STATISTICAL ANALYSIS 14

13 DATA MANAGEMENT 14

14 HANDLING OF RECORDS 14

14.1 Procedures for the recording, processing and handling of study documentation 14

14.2 Records to be retained by Investigator 14

14.3 Retention of records after completion of the study 14

14.4 Final report 14

15 OPERATIONAL MATTERS 15

15.1 Monitoring 15

16 Changes to the protocol 15

16.1 Protocol amendment 15

16.2 Deviation from protocol 15

17 Animal ethics 15

18 QUALITY ASSURANCE 15

19 REFERENCES 15

20 SUPPLEMENTS 16

20.1 List of Working Instructions (WIs) 16

20.2 List of Data Capture Forms (DCFs) 16

**LIST OF TABLES**

[Table 1: Collaborating researchers and their affiliation 6](#_Toc454461777)

[Table 2: Ecological infestation/infection statistics that may be used – calculation of prevalence (Bush et al. 1997) will be mandatory 12](#_Toc454461778)

**LIST OF APPENDICES**

**Appendix A**: Working Instruction: Weight and Body Condition Scoring 18

**Appendix B** Working Instruction: Tick counts and collections from live animals (smallholder farms).............................. 21

**Appendix C**  Working Instruction: Faecal collection from live animals, diagnostic faecal flotation, sedimentation, larval culture and larval egg count (smallholder farms)………………………………………………………………………………….................23

**Appendix D** Working Instruction: collection of helminths (Necropsy at abattoir)....................30

**Appendix E**  Working Instruction: Blood specimen collection and processing (smallholder farms)…………………………………………………………........................................….........32

**Appendix F** Packaging and transportation instructions.............................................................33

**GLOSSARY OF ABBREVIATIONS**

| BMGF | Bill and Melinda Gates Foundation |
| --- | --- |
| DCF | Data Capture Form |
| EPG | Eggs Per Gram |
| FTA® card | Flinders Technical Associates card |
| GCP | Good Clinical Practice |
| GLP | Good Laboratory Practice |
| ID | Identification |
| PPE | Personal Protection Equipment |
| PCR | Polymerase Chain Reaction |
| SOP | Standard Operating Procedure |

1. TITLE

Survey of ecto- and endoparasite taxa impacting on small-scale rural livestock farmers in Benin.

- 1. Project reference

CV 16/337

1. STUDY CONTACTS
   1. Sponsor Company

ClinVet International (Pty) Ltd

PO Box 11186

Universitas 9321

Bloemfontein

South Africa

- 1. Sponsor Representative

Prof M Madder (PhD)

Clinglobal Limited

Dagpauwoog 12

4904 ZP Oosterhout

The Netherlands

Mobile: +0031 63 95 63 600

E-mail: maxime.madder@clinglobal.com

- 1. Investigator

Dr SB Adehan (MSc)
National Institute of Agricultural Research (INRAB)
01 BP 884
Cotonou
Benin
Telephone: +229 96785061
Facsimile:
E-mail: adehankarim@yahoo.fr

- 1. Collaborating researchers and departments of affiliation

Researchers and departments collaborating with the Investigator on this project are tabulated below:

**Table 1: Collaborating researchers and their affiliation**

| **Researcher** | **Affiliation** |
| --- | --- |
| GBEGO TOSSA Isidore | Zootechnical, Veterinary and Halieutic Research laboratory (LRZVH) |
| FAROUGOU Souaïbou | Polytechnic High School of Abomey-Calavi/UAC/Epac |
| ADEHAN Safiou B. | Zootechnical, Veterinary and Halieutic Research laboratory (LRZVH) & Polytechnic school of Abomey-Calavi UAC |
| AYENA Jean | Ministry of Agriculture, Livestock and Fisheries, Agriculrural center of Djougou |
| GBAGUIDI Michel | Ministry of Secondary Education/Sékou Agricultural school&Zootechnical, Veterinary and Halieutic Research Laboratory (LRZVH) |
| DOSSOUMOU Aquilas | Ministry of Agriculture, Livestock and Fisheries/Agriculrural center of Savè&District office ofSavè |
| BADAROU O. Kadidjatoulaï | Zootechnical, Veterinary and Halieutic Research laboratory (LRZVH) |
| HOUMBADE Emmanuel | Ministry of Agriculture, Livestock and Fisheries/Agriculrural center of Djougou |
| TOHOZIN Rogatien | Ministry of Agriculture, Livestock and Fisheries/Pafilav Laboratory |
| CHABI ALOBA G. Désiré | Ministry of Agriculture, Livestock and Fisheries/ Agriculrural center of Savalou& Laboratory of Pafilav |

1. OBJECTIVE(S) OF THE STUDY

The objective of the study is to identify the most economically important ecto- and endoparasites affecting the smallholder livestock production systems in Benin, so as to provide a solid scientific basis for their future control.

Breakdown of specific objectives is as follows:

i. Establish the current baseline prevalences of the most important ecto- and endoparasite species that are of greatest importance to the smallholder livestock production systems in Benin;

ii. Collect strains of the most important ecto- and endoparasite taxa and establish pure colonies in the laboratory (i.e. establish laboratory strains);

1. BACKGROUND AND JUSTIFICATION FOR STUDY

The economy of Western Africa is based mainly on the activities of the rural sector. In Benin the livestock sector accounts for approximately 6% of the GDP ([www.fao.org](http://www.fao.org)) and more than 70% of the population works in this sector (Tijani et al., 2006). Livestock is important in livelihood strategies of the poor. Such strategies include savings, insurance, security, accumulation and diversification of assets, as well as social and cultural functions. Livestock production in 2013 in Benin are estimated to include over two million bovines, two and a half million small ruminants, almost half a million pigs and almost 20 million chickens (Ministry of Agriculture, 2013; www.fao.org). This is not enough to cover the country’s needs in animal protein, particularly in meat, milk and eggs (Ministry of Agriculture, 2013; www.fao.org). However, many health constraints are encountered in the sector, among which infestations by ectoparasites (predominantly ticks) and endoparasites (predominantly helminths) cause significant economic losses (De Castrates et al., 1993; Ogden et al., 2004; Jonsson, 2006; Bianchin et al., 2007).

There are two general types of traditional cattle production in Benin; (i) sedentary production in the Guinean region, which accounts for about 20% of the national herd, and (ii) transhumant production (seasonal movement of people with their livestock between fixed summer and winter pastures), which accounts for the other 80% (Atchy, 1976). In cattle the most important parasitic diseases are Babesiosis and Trypanosomosis. With the introduction of *Rhipicephalus microplus* in 2006 in Benin, the epidemiology of Babesiosis might have changed significantly (De Clercq et al., 2012) and the control of ticks became problematic. High degrees of resistance have been observed in most of the country resulting in inappropriate use of acaricides (Madder et al., 2011; De Clercq et al., 2012) and high tick numbers that lead to production losses. Not much information is available on the importance and impact of tick-borne diseases of small ruminants. Areas of interest for further research would certainly include impact of the introduction of *R. microplus*, its distribution, expansion and resistance to acaricides. Futhermore it is recommended to determine the effect of the expansion of this tick on the epidemiology and effect of Babesiosis.

The impact of helminth diseases in Benin is, as in most developing countries, highly dependable upon infection rate and number of infective stages (prevalence and abundance), the predilection site of the parasite, population density and the immune response of the host. For these reasons an estimation of the economic relevance of helminth infestations is hard to achieve. In cattle, sheep and goats most emphasis is seen in the field of losses as a result of liver condemnation.

1. SCHEDULE OF EVENTS
   1. Proposed dates

| First sampling date (Visit 1): | August 2016. |
| --- | --- |
| Second sampling date (Visit 2): | November 2017 |
| Third sampling date (Visit 3): | April 2017 |
| Final samling date (Visit 4): | June 2017. |
| Report date: | August 2017. |

1. STUDY DESIGN
   1. Type of study and overall study design

This is a multi site field survey to establish the current baseline prevalences of the most important ecto- and endoparasite species that are of greatest importance to the smallholder livestock production systems in Benin. Sampling sites are located in 10 localities (districts), belonging to two Departments (Donga and Zou, respectively). The concerned localities with related Departments are listed below:

- Districts of Djougou, N’dali, OuassaPehunco, Bassila and Ouaké belong to the Donga Department and are considered as a pool of sampled sites, denoted “Pool 1”.
- Districts of Abomey, Djidja, Ouinhi, Zangnannando and Zogbodomey, belonging to the Zou Department and are considered as a pool of sampled sites, denoted “Pool 2”.

Sampling sites consist of smallholder farmer settlements, taking into account herds that are mainly sedentary and located in the listed localities. These farmers are considered to belong to communities of poor resource stockbreeders.

The survey will be conducted over a period of 18 months (July 2016 to December 2017). Field work will consist of eight trips, namely four trips for sample sites of Pool 1 and four trips for sample sites of Pool 2. The same sampling periods are thus to be considered for both pools, resulting in four sampling periods (quaternary sampling). Each visit will include sampling at both the smallholder herds as well as selected abattoirs.

The first visit to both pools will take place in August 2016 (Visit 1), during the peak of the rainy season in the North (Pool 1 locality). This may potentially, but not necessarily, correspond to the early dry season period in central Benin (Pool 2 locality). This is because the central region is a transition climatic area that may also be subject to rainfall in August during some years. The second visit (both pools) is planned for November 2016 (Visit 2) at the early dry season, the third for the start of the rainy season in April 2017 (Visit 3) and finally the fourth visit during the rainy season in June 2017 (Visit 4).

Each trip duration is expected to be seven days, but may vary depending on logistical challenges encountered. Such trips will include the outward journey, home journey and assessments (see Section 9) performed at selected farms located at each study site. The actions to be conducted during each site visit are summarized below:

| **Visit** | **Action** |
| --- | --- |
| 1, 2, 3 & 4 | blood samples on FTA® cards |
|  | tick collections and sampling |
|  | faecal samples for faecal egg counts in eggs per gram of faeces (EPG) |
|  | Complete questionaire |
|  | Helminth recovery at abbotoir (only once or twice) |

- 1. Experimental unit and justification

The experimental unit is individual cattle. Cattle will be used since it represents the most important production animal to small scale rural farmers.

- 1. Sample size and justification
     1. Smallholder herds

Not less than five animals per herd will be sampled. Ideally 120 cattle will be sampled for ticks (half body collections), helminths (faecal samples) and haemoparasites (blood specimen sampling). This number is based on the number of animals that can be handled by the sampling teams during one sampling day, namely four animals per hour (24 animals per day) resulting in 120 animals per five day sampling period. This translates into 4 x 120 = 480 samples from each of the two different localities (Pool 1 and Pool 2).

The total number of samples will thus be:

- 960 dried DNA blood samples on FTA® cards;
- 960 tick collections in ethanol; a representative number of engorged ticks for laboratory strain establishment purposes;
- 960 faecal samples for faecal egg counts in eggs per gram of faeces (EPB) and subsequent larval development.
  - 1. Abbottoir

The number of cattle to be processed will depend on logistical constraints, such as abattoir schedules and availability of animals for slaughter during site visits. One or two slaughter houses will be identified in each of the two localities. In each locality at least 20 animals should be checked and samples taken of the abomasum, intestine and liver.

Counts and collections will include helminth recovery.

1. **ANIMAL SELECTION AND IDENTIFICATION**

| Species: | *Bovine.* |
| --- | --- |
| Breed: | No restrictions |
| Localities: | Districts of Djougou, N’dali, OuassaPehunco, Bassila and Ouaké belong to the Donga Department and are considered as a pool of sampled sites, denoted “Pool 1”.  Districts of Abomey, Djidja, Ouinhi, Zangnannando and Zogbodomey, belonging to the Zou Department and are considered as a pool of sampled sites, denoted “Pool 2”. |
| Number to be sampled per visit: | At least 120 cattle per locality (pool). |
| Age: | ≥ 1 – 2 years |
| Gender: | Not restricted. |
| Body weight: | Not restricted. |
| Health status: | Not restricted. |
| Identification method: | Animals don’t need to be identifiable |

1. **INCLUSION CRITERIA**

Cattle will be included in this survey if:

- they are 1 to 2 years old;
- they have not been treated with a topical or systemic acaricide/insecticide/anthelmintic for the last 2 weeks;
- they are not excessively fractious in that they pose a danger to themselves or study site personnel.

If any animal does not comply with the above mentioned criteria, it will not be included.

1. **ENVIRONMENTAL MONITORING**
   1. **Temperature**

Minimum, maximum and average temperatures will be obtained from the nearest weather station.

- 1. **Rainfall**

Minimum, maximum and average rainfall data will be obtained from the nearest weather station.

1. ASSESSMENTS
   1. Weigh estimation and body condition scoring

The weight of cattle from small holder herds to be sampled for parasites will be estimated using a RONDO^®^ tape according to the manafacturers recommendations. Body conditioning scoring will be also be conducted for these animals according to working instructions (WI) in Appendix A.

- 1. Tick counts and sampling
     1. Sampling from smallholder farms

Smallholder herd tick counts and collections from live animals will be performed as described in the relevant Working Instruction (WI) in Appendix B. Samples will be uniquely identified as follows: samples will be identified by a unique code consisting of the country code (Benin: BN), Department code (Donga: D, Zou: Z), farm number, a 3-digit progressive number and sample type (ticks: T), e.g., the first animal on the first farm visited in the Department of Zou will be allocated number BN-Z-1-001-T. The second animal on the same farm BN-Z-1-002-T, etc. The date of collection will also be written on each of the samples.

Preserved Samples will be transferred to Zootechnical, Veterinary and Halieutic Research laboratory for tick identification and enumeration. Tick identification is done based on morphology using a stereoscope (80-fold magnification) and a microscope (100 to 200-fold magnification to better visualize the hypostome dentition of blue ticks). Only adult specimens are identified up to species level using both taxonomic descriptions (Walker et al. 2003) and morphological keys (Madder 2012a, b). The identification and confirmation of *R. microplus* in areas where the ticks was never found before, can be done molecularly using a PCR–RFLP test (Lempereur et al. 2010).

- 1. Faecel flotations/sedimentations and helminth sampling
     1. Sampling from smallholder farms

A diagnostic faecal flotation and sedimentation method will be used to identify eggs in faecal samples. Quantification of eggs is performed using the McMaster method. In addition larvae will be cultured and identified. The methods to be used are described in the relevant WI in Appendix C. Samples will be uniguely identified as follows: samples will be identified by a unique code consisting of the country code (Benin: BN), Department code (Donga: D, Zou: Z), farm number, a 3-digit progressive number and sample type (faecal material: F), e.g., the first animal on the first farm visited in the Department of Zou will be allocated number BN-Z-1-001-F. The second animal on the same farm BN-Z-1-002-F, etc. The date will also be written on each of the samples. Eggs samples that have hatched and stored as subsequent samples, will be given an additional 1-digit code, e.g. eggs from sample BN-Z-F-1-001 that gave rise to larvae will be labelled as BN-Z-1-001-F-1. The date of collection will also be written on the samples.

All faecal samples will be transferred to Zootechnical, Veterinary and Halieutic Research laboratory for identification . Nematode and Cestode eggs are collected and detected using the floatation test as described in the relevant Working Instruction (WI) in Appendix D or and The RVC/FAO Guide to Veterinary Diagnostic Parasitology (<http://www.rvc.ac.uk/review/parasitology/Flotation/General.htm>). For Trematode eggs the faecal sedimentation test is used as described in the relevant Working Instruction (WI) in Appendix D or and The RVC/FAO Guide to Veterinary Diagnostic Parasitology (http://www.rvc.ac.uk/review/parasitology/FaecalSedimentation/Purpose.htm).

- - 1. Sampling from abbotoirs

A complete helminth recovery assessment will be performed according to procedures described in the relevant WI in Appendix D. Preserved samples will be transferred to Zootechnical, Veterinary and Halieutic Research laboratory for identification and enumeration. Samples will be uniquely identified as follows: samples will be identified by a unique code consisting of the country code (Benin: BN), Department code (Donga: D, Zou: Z), abattoir number, a 3-digit progressive number and sample type (abomasum: A, small/large intestine: I, liver: L),, e.g., the sample of the abomasum of the first animal on the first abattoir visited in the Department of Zou will be allocated number BN-Z-1-001-A. The abomasum sample of the second animal in the same abattoir BN-Z-1-002-A, etc. The date of collection will also be written on each of the samples.

A short questionnaire will be completed for each of the animals slaughtered as described in section 11.5 below.

- 1. Blood collection and testing for haemoparasites

Blood samples (approximately 125 μl) will be collected from the middle ear vein using a capillary tube. Samples will be applied onto Whatman® FTA® cards, air-dried and labelled appropriately and packed in foil pouches with a silica gel, prior to DNA analysis (Muhanguzi et al, 2014).

Testing will be performed to assess presence of Babesiosis and Trypanosomosis. DNA analysis will be according to the method described by Muhanguzi et al. (2014).

Abattoir assessment: Blood specimens will not be collected from animals at abattoirs.

Samples will be identified by a unique code consisting of the country code (Benin: BN), Department code (Donga: D, Zou: Z), farm number, a 3-digit progressive number and sample type (blood: B), e.g., the first animal on the first farm visited in the Department of Zou will be allocated number BN-Z-1-001-B. The second animal on the same farm BN-Z-1-002-B, etc. The date of collection will also be written on each of the samples.

- 1. Assessment of demographic, socio-economic parameters and husbandry practices

Structured questionnaires will be administered to the owner of each herd so as to gather information about the relative importance of livestock to such a household as well as other demographic and socio-economic parameters and also to get an idea of the basic husbandry practices.

A similar questionnaire will be created for abattoirs, so as to also gather information on economic impact of parasites in terms of condemnation of carcasses or organs (such as liver).

These questionnaires are provided in DCF 10 and 11.

- 1. Laboratory strain establishment
     1. Tick strain colony establishment

Engorged females of representative tick species will be shipped to Clinvet International (Morocco site) according to a material transfer agreement between Zootechnical, Veterinary and Halieutic Research laboratory (LRZVH) in Benin and Clinvet lnternational. The shipped specimens will be used to establish pure tick laboratory strains.

- - 1. Helminth strain colony establishment

Representative helminth species (infective larvae harvested following coproculture) will be shipped to Clinvet International (Morocco) according to a material transfer agreement between Zootechnical, Veterinary and Halieutic Research laboratory (LRZVH) in Benin and Clinvet lnternational. The shipped specimens will be used to establish pure helminth laboratory strains that will be used for laboratory evaluation of the effectiveness of different pharmaceutical and biological products for control of the most important helminths in Benin.

Working Instruction (WI) on packaging and transportation of biological material according to the IATA guidelines is given in Appendix F.

1. STATISTICAL ANALYSIS

Only descriptive analysis will be given for the obtained results.

1. DATA MANAGEMENT

Data captured on the Data Capture Forms (DCFs) will be double data entered into a SAS database by two independent typists. The SAS database is designed to match the structure of the study specific DCFs, with values formatted appropriately.

A PROC COMPARE report from SAS will be used to identify any discrepancies that may have occurred between first and second data entry. Discrepancies will be resolved to reflect the correct value as per the study documentation.

1. HANDLING OF RECORDS
   1. Procedures for the recording, processing and handling of study documentation

The individual entering the data will record all data generated during the course of the study directly, promptly, accurately and legibly in black ink. These entries will be signed or initialled and dated. Any change in study documentation will be made in such a manner as to not obscure the previous entry. Error codes will be used to indicate the reason for the change. Changes will be signed or initialled and dated by the individual making the change. Data subjected to statistical analysis will be handled according to the procedures set out in Section 13.

In addition to the DCFs that are included as protocol appendices, other additional forms and/or computer printouts may be included at the discretion of the Investigator.

- 1. Records to be retained by Investigator

The records to be kept include, but are not limited to the following:

• personnel list, signatures and duties;

• all source data forms;

• approved protocol;

• protocol amendments and deviations;

• Investigator’s report and report amendments;

• study related correspondence.

- 1. Retention of records after completion of the study

Original study documentation, together with the final report, will be handed over to the Sponsor after completion of the study. A certified copy of the study documentation and report will be retained in the historical archives of Clinvet for a period of five years.

- 1. Final report

The report will be written by the Sponsor representative and Investigator in conjunction with other qualified personnel appointed, if required. The report will describe all materials and methods used and results.

1. OPERATIONAL MATTERS
   1. Monitoring

Monitoring will be performed by the sponsor representative. Visits will be made during at least one of the visits. Timing will be agreed upon in advance. Audits of all data related to the trial will be conducted by the Monitor.

The audits, as well as examination of all source data (original records, laboratory notebooks, et cetera), may be conducted at any time during the study or when the animal phase of the study is concluded.

1. Changes to the protocol
   1. Protocol amendment

Protocol amendments (changes made prior to protocol implementation) will be numbered sequentially, approved and signed by the Investigator and Sponsor and filed with the protocol. However, if other personnel (such as the Statistician) also signed the protocol, they must sign the amendment as well if the changes implemented specifically apply to sections directly relevant to them.

- 1. Deviation from protocol

Deviations from protocol (report of any action not in accord with the protocol) will be numbered sequentially and filed with the study documentation. The Investigator will sign the deviation.

1. Animal ethics

An application to the Institutional Animal Care and Use Committee (IACUC) will be submitted for approval to conduct the study. Upon approval, a certificate of approval will be issued.

1. QUALITY ASSURANCE

The Quality Assurance unit of Clinvet will audit the protocol, the final report and the study documentation. A Quality Assurance statement will be appended to the final report.

1. REFERENCES

Atchy, A. A. 1976. Contribution a l'etude de la transhumance en Republique populaire du Benin (Doctoral dissertation, Universite de Dakar.)

Bianchin, A., De Luca, A., & Caminiti, A. 2007. Postoperative vomiting reduction after laparoscopic cholecystectomy with single dose of dexamethasone. Minerva Anestesiologica, 73(6), 343-346.

De Clercq E. M., Vanwambeke S. O., Sungirai M., Adehan S., Lokossou R. et Madder M. 2012. Geographic distribution of the invasive cattle tick *Rhipicephalus microplus*, a country-wide survey in Benin. ExpApplAcarol.Dec;58(4):441-52. DOI 10.1007/s10493-012-9587-0.

Jönsson, H., Heisler, M. G., Shapiro, B. E., Meyerowitz, E. M., & Mjolsness, E. 2006. An auxin-drivenpolarized transport model for phyllotaxis. Proceedings of the National Academy of Sciences, 103(5), 1633-1638.

Lempereur L, Geysen D, Madder M (2010) Development and validation of a PCR–RFLP test to identify African Rhipicephalus (Boophilus) ticks. Acta Trop 114:55–58

Madder M., Thys E., Achi L., Toure A., De Deken R. 2011. *Rhipicephalus (Boophilus) microplus*: a most successful invasive tick species in West-Africa. ExpApplAcarol 53(2):139–145.

Madder M., 2012a. I-Spot key for the identification of R. Boophilus females. http://www.ispot.org.uk/webkeys/keyintroduction.jsp?selectedKey=webkeys/Rhipicephalus(Boophilus)_females.0.1

Madder M., 2012b. I-Spot keys for the identification of R. Boophilus males. http://www.ispotnature.org/webkeys/keyintroduction.jsp?selectedKey=webkeys/Rhipicephalus(Boophilus)_males.0.2

Margolis, L., Esch, G.W., Holmes, J.C., Kuris, A.M. and Schad, G.A. 1982. The use of ecological terms in Parasitology (report of an ad hoc committee of the American Society of Parasitologists). The Journal of Parasitology: 68(1), 131-133.

Muhanguzi, D., Picozzi, K., Hatendorf, J., Thrusfield, M., Welburn, S. C., Kabasa, J. D., &Waiswa, C. 2014. Prevalence and spatial distribution of *Theileria parva* in cattle under crop livestock farming systems in Tororo District, Eastern Uganda. ParasitVectors, 7(1), 91.

Ogden N. H ., Swai E., Beuchamp G., Karimuribo E., Fitzpatrick J. L., Bryant M. J., Ambarage D., et French N. P. 2004. Risk factors for tick attachment to smallholder dairy cattle in Tanzania. Prev. vet. med., 67:157-170

Tijani, M. A. 2006. Difficultés de communication orale: enquête sur les stratégies de communication des apprenants nigérians de français en situation exolingue (Doctoral dissertation, Besançon).

1. SUPPLEMENTS
   1. List of Working Instructions (WIs)

The following WIs are provided in Appendix A to F:

**Smallholder farm asessments:**

- Tick counts and collections from live animals (smallholder farms);
- Blood specimen collection and processing (smallholder farms);
- Faecal collection from live animals, diagnostic faecal flotation, sedimentation, larval culture and larval egg count (smallholder farms).

**Abattoir assessments:**

- Collection of ectoparasites (half body tick counts and collections during necropsy at abattoir);
- Collection of helminths (necropsy at abattoir).

Transportation of biological material (IATA regulations)

- Biological sample preparation
- Packaging of samples

Note that this list is not to be considered exhaustive and that other Standard Operating Procedures (SOPs) or working instructions may also be applicable and may be consulted by study personnel as required.

- 1. List of Data Capture Forms (DCFs)

DCFs that will be used in this study include, but may not necessarily be limited to, those listed below (included in Appendix G).

[Note: To be designed and adapted based on WIs once compiled]:

- DCF1 Animal details and inclusion
- DCF2 tick identification
- DCF3 helminth eggs identification: sedimentation
- DCF4 helminth eggs identification: larval culture
- DCF5 helminth eggs identification: floatation
- DCF6 helminth eggs identification: faecal egg count
- DCF7 helminth identification: adult worms
- DCF8 abattoir sampling
- DCF9 Sample collection and half body tick counts
- DCF 10: Questionnaire: demographic and socio-economic parameters
- DCF 11: Questionnaire: animal health interventions

Adjustments to these forms, such as typing animal identification (ID) information or deleting/adding rows as required, will not require a protocol amendment. The Investigator may also include additional *ad hoc* forms as required, examples of which will be included in the study documentation.

**Appendix A**: Working Instruction: Weight and Body Condition Scoring

Body weight scoring

Body weight of the cattle that are included in the study are measured using the Rondo^®^ measuring tape for cattle.

Body condition scoring

Body condition scores are excellent indicators of the nutritional status in beef cows. Ideal liveweight varies from cow to cow whereas ideal body condition (BCS 5-6) is the same for all cows. Also, body condition can be measured in the field without gathering or working cattle.

Body condition scores are numbers used to estimate energy reserves in the form of fat and muscle of beef cows. BCS ranges from 1 to 9, with a score of 1 being extremely thin and 9 being very obese. Areas such as the back, tail head, pins, hooks, ribs, and brisket of beef cattle can be used to determine BCS (Figure 1).


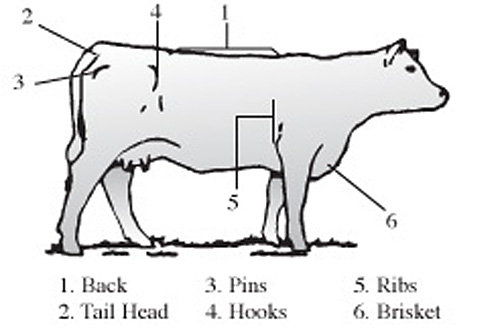
Figure 1. Areas useful for visually determining BCS in beef cows. (Oklahoma State University)

A cow in 'thin' condition (BCS 1-4) is angular and bony with minimal fat over the backbone, ribs, hooks, and pins. There is no visible fat around the tail head or brisket. A cow in 'ideal' condition (BCS 5-7) has a good overall appearance. A cow with a BCS of 5 has visible hips, although there is some fat over the hooks and pins and the backbone is no longer visible. Cows with BCS of 6 or 7 become fleshy and the ribs are no longer visible. There is also fat around the tail head and in the brisket. An over-conditioned cow (BCS 8-9) is smooth and boxy with bone structure hidden from sight or touch. She may have large protruding fat deposits (pones) around the tail head and on the pin bones. Be aware that gut fill due to rumen contents or pregnancy can change the appearance of moderately fleshy cows, especially over the ribs or in front of the hooks. Visual indicators of each BCS are listed in Table 1, and example photos of BCS 1-9 are illustrated in photos 1 through 9.

| **Table 1. Reference table for body condition scores.** | | | | | | | | | |
| --- | --- | --- | --- | --- | --- | --- | --- | --- | --- |
| **Reference point** | **Body Condition Scores** | | | | | | | | |
|  | **1** | **2** | **3** | **4** | **5** | **6** | **7** | **8** | **9** |
| Physically weak | yes | no | no | no | no | no | no | no | no |
| Muscle atrophy | yes | yes | slight | no | no | no | no | no | no |
| Outline of spine visible | yes | yes | yes | slight | no | no | no | no | no |
| Outline of ribs visible | all | all | all | 3-5 | 1-2 | 0 | 0 | 0 | 0 |
| Outline of hip & pin bones visible | yes | yes | yes | yes | yes | yes | slight | no | no |
| Fat in brisket and flanks | no | no | no | no | no | some | full | full | extreme |
| Fat udder & patchy fat around tail head | no | no | no | no | no | no | slight | yes | extreme |
| *(Modified from Pruitt, 1994.)* | | | | | | | | | |

Long hair can often make it difficult to correctly evaluate the body condition score of a beef cow or heifer. When the hair on the cow is long, palpating the specific areas of fat deposition is particularly important, as shown in Figure 2. Cows should be palpated over the back, ribs, and over the horizontal processes of the backbone (edge of loin). 'Thin' cows will have a sharper feel in these areas than cows with moderate or fat body conditions.


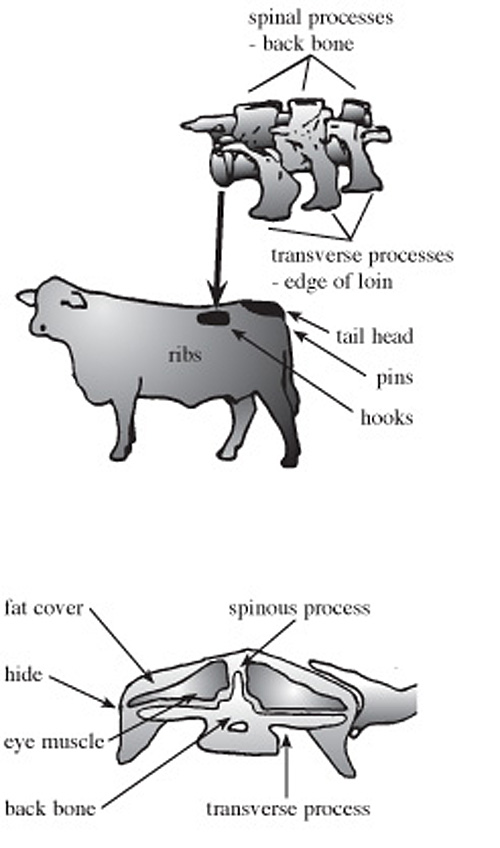


Figure 2. Specific anatomical areas used in determining BCS in beef cows. (Adapted from Herd and Sprott, 1986.)

It is important to be aware that the breed of beef cow can have a strong influence on where body fat is deposited. For example, *Bos taurus* breeds and crossbreds will show a more uniform distribution of fat across the ribs, whereas *Bos indicus* cattle may have very little fat over the ribs but will deposit fat over the hooks and pin bones.

**Appendix B** Working Instruction: Tick counts and collections from live animals (smallholder farms)

**Objectives**

The sampling at the different study sites has the following three objectives:

1. Complete gaps of knowledge identified in the desk review (cross-sectional tick survey)
2. Collect live ticks to determine acaricide resistance by Shaw Larval Immersion Test (phase 2)
3. Isolate ectoparasites to test intervention strategies in phase 2 of the project

**Collection of ticks**

Ticks have to be collected for the cross-sectional study, resistance study and also for ectoparasite isolation. For the resistance and isolation, ticks should remain alive and transferred to the laboratory for future studies or exported to ClinVet Morocco.

**Cross-sectional survey (ticks on alcohol)**

To collect ticks from cattle, animals will be restrained, put in a crush-pen or put down if the situation allows it. Half body sampling will be performed on 5 different pre-dilection sites: (i) the inner and outer fore-legs, hind-legs and abdomen, (ii) tail and anal area, (iii) head and neck, (iv) lateral area and dorsal area shoulders to tail base and (v) ears. The ticks, males, females, nymphs and possibly larvae, are removed using forceps and for ticks of the genus *Rhipicephalus (Boophilus)* attention should be paid to collect male ticks as well as they are easier to be identified. Male ticks are always attached below an engorging female. When the female is lifted, the males become visible and should be taken first. The collection is performed for about 15 minutes in total from all the pre-dilection sites. For heavily infested animals, the remaining ticks that couldn’t be collected are counted and recorded on the data capture form (DCF 2). Attention is paid to the different genera.

Collected ticks are stored in plastic vials containing 70% alcohol. Each vial should contain a pencil written label with sample ID as recorded on the DCF 2.

**Live ticks**

Live ticks should be collected with forceps but caution should be given not to damage the ticks or its mouthparts.

1. For Shaw Larval Immersion Tests (LIT), ± 50 engorged females are collected per site from a number of highly infected animals and stored in plastic vials with perforated lids. For transport vials should be stored in a cool box and not exposed to high temperatures or direct sunlight. Humidity can be regulated by putting humidified filter paper at the bottom of the cool box. Once in the laboratory, ticks are identified and stored per species and per animal in glass Erlenmeyer flasks closed with cotton wool. These labelled flasks are stored at 25°C and 87%RH. Predicted hatch date (PHD)(estimated to be 40 days from collection date) is added to the label. Actual hatching date is recorded when approximately 75% of the larvae have hatched.
2. For isolation of ectoparasites, live ticks should be stored similar as described above. Transport of the live ticks to the laboratory of ClinVet Morocco requires the ticks to be stored and shipped according to the IATA guidelines.

**Sampling strategy**

**Cross-sectional survey**

Per country, two sampling locations are identified described in the “Project registration” document signed by each country.

In each location, preferably 120 cattle (age ranging between one and two year) will be sampled for ticks (half body collections). This number is based on the number of animals that can be handled during one sampling day: 4 animals per hour, 24 per day and 120 per five days sampling period. This means 4 x 120 = 480 samples on each of the two different sites. In total 960 animals sampled per country.

Animals that qualify for the study should fulfil the inclusion criteria: (i) age between 1 and 2 years, (ii) not been treated with a topical or systemic acaricide/insecticide/anthelmintic for the last 2 weeks, (iii) not fractious in that they pose danger to themselves or personnel.

**Live ticks for LIT and isolation**

Per country, 5 farms will be visited in each of the two study sites, meaning 10 sites per country. Ticks collected at one farm will be named an isolate. Ticks can be collected from a number of animals visibly highly infected.

Collection takes place before the cattle are dipped, sprayed or treated with an acaricide. Only engorged females greater than 4mm are collected as this is the minimum size of engorged female correlated with ability to lay eggs. For identification it is recommended to collect male ticks as well as they are easier to be identified.

**Appendix C**  Working Instruction: Faecal collection from live animals, diagnostic faecal flotation, sedimentation, larval culture and larval egg count (smallholder farms)

**Collection of faecal material**

Faecal samples for parasitological examination should preferably collected from the rectum. Appropriate disposable gloves should be worn. Collection from large animals can be more easily accomplished than from smaller animals.

**Collection procedure**

1. Put on a clean glove. Apply a nickel size amount of water or water-based lubricant to the glove.

2. Insert hand into the rectum of the animal, remove faecal matter.

4. Peel the glove off your hand keeping the faecal sample encased within it.

5. Squeeze as much air as possible out of the glove. Twist the wrist portion of the glove and fasten with a label (see protocol for labelling) making sure the label sticks to itself, as it won’t stick to the glove. You can also twist and tie off the glove and label the glove itself with an indelible marker.

Store the sample in the refrigerator until it can be analyzed (the sooner the better, but samples can be stored in the refrigerator for a week). If you are collecting many samples at one time, have a cooler with ice on hand to keep the samples cool until you can get them into a refrigerator.

Samples can be collected from the ground if the animal is seen defaecating. Faeces collected in this way must be fresh to be suitable for parasitological examination.

**Diagnostic Flotation technique**

The simple test tube flotation technique is a qualitative test for the detection of nematode and cestode eggs. This is a useful method to use in preliminary surveys to establish which parasite groups are present. Eggs are separated from faecal material and concentrated by a flotation fluid of an appropriate specific gravity.

**Equipment List**

- Two beakers or plastic containers
- Tea strainer or double layer cheesecloth
- Measuring cylinder or container graded by volume
- Fork, tongue blades or stirring rod
- Test tube
- Test tube rack
- Microscope
- Microscope slides and coverslips
- Balance or teaspoon
- Flotation fluid
  - For genral purpose
    - specific gravity: 1.18 - 1.20
    - sodium chloride: 400g/1000ml
    - stir thoroughly before use
    - may distort eggs
  - For culturing of eggs
    - specific gravity: 1.27
    - sugar (sucrose) 454g/355ml
    - 2ml of 37% formaldehyde to prevent mould growth
    - stir solution before using
    - saturation is indicated by the presence of sugar cristals, if not present, add sugar and stir until sugar doesn’t dissolve any more

**Procedure**

- Weigh or measure using a precalibrated teaspoon approximately 3g of faeces and put into container 1.
- Pour 50 ml of flotation fluid into container 1.
- Stir or mix faeces and flotation fluid thoroughly with a tongue blade or fork.
- Pour the faecal suspension through a tea strainer or double layer of cheesecloth into container 2.
- Pour the faecal suspension into test tube supported in a stand or rack from container 2.
- The test tube is gently topped off with the suspension leaving a convex meniscus at the top of the tube.
- Carefully place a coverslip on top of the test tube.
- Leave the test tube to stand for 20 minutes.
- Carefully lift the coverslip off the test tube together with the drop of fluid adhering to it.
- Place the coverlip on a clean slide.
- Examine using a compound microscope at 10 x 10 magnification.

**Diagnostic Sedimentation technique**

The sedimentation technique is a qualitative method for detecting trematode eggs in faeces. The majority of trematode eggs are too large and heavy to float reliably in the flotation fluids normally used for nematode eggs. They do however sink rapidly to the bottom of a faecal/water suspension and this is the basis of the faecal sedimentation technique.

**Equipment List**

- Two beakers or plastic containers
- Tea strainer or double layer of cheesecloth
- Measuring cylinder
- Fork, tongue blades or stirring rod
- Test tubes
- Test tube rack
- Methylene blue 1% solution or Malachite green 1% solution
- Microscope slides and coverslips
- Pipettes
- Balance or calibrated teaspoon
- Microscope

**Procedure**

- Weigh or measure approximately 3 g of faeces into container 1.
- Pour 40-50 ml of tap water into container 1.
- Mix faeces and water thoroughly.
- Filter the suspension through a tea strainer or double-layer of cheese cloth into container 2.
- Pour the filtered material into a test tube.
- Remove the supernatant with a pipette very carefully.
- Re-suspend the sediment in 5ml of water.
- Allow to sediment for 5 minutes.
- Discard the supernatant carefully.
- Stain the sediment by adding one drop of methylene blue or malachite green.
- The dyes stain the faecal particles a deep blue or green leaving the trematode eggs unstained.
- Transfer a small drop of the stained sediment to a microscope slide using a pipette.
- Cover droplet with a coverslip.
- Examine under a microscope at 10 x 10 magnification.
- Repeat until all the sediment has been examined.
- Alternatively, pour the whole amount into a Petri dish and examine methodically under a stereo-microscope.

**Identification of eggs**

Eggs can be identified using the morphological features as described by http://www.rvc.ac.uk/review/parasitology/RuminantEggs/Common.htm

**Larval culture**

This procedure is for diagnosing the third stage larvae of strongyle nematodes present in faeces which are recovered using the Baermann technique.

**Equipment List**

- Fork, spoon, tongue depressor, spatula
- Water
- Jars, containers
- Charcoal (dried, sterile bovine faeces may be used if charcoal is not available. To use bovine faeces heat to 70°C. This sterilizes the faeces and removes any helminth eggs. Once sterilized the sample is ground to a fine powder.)
- Incubator (if available)

**Procedure**

- Break up faeces in a container a spatula or other stirring device.
- Faeces should be moist and crumbly. If the faeces are too dry add water until correct consistency is obtained. If the faeces are too wet, add charcoal or sterile bovine faeces until correct consistency is obtained.
- Transfer the mixture to jars or containers.
- Leave the culture at room temperature for 14-21 days when larvae should have reached infective stage. Add water to cultures regularly if mixture is getting too dry, approximately every 1-2 days.
- If an incubator is available, the culture can be placed at 27^o^C and left for 7-10 days. Add water to cultures regularly if mixture is getting too dry, approximately every 1-2 days.
- The larvae are recovered by [Baermann technique](http://www.rvc.ac.uk/review/parasitology/Baermann/Purpose.htm).

**Baermann technique**

The Baermann technique is used to separate larvae from faecal material and diagnosing of e.g. lungworm infection or identification of third stage larvae (L_3_) from faecal culture. This technique is based on the active migration or movement of larvae.

**Equipment List**

| - Funnel – size according to need - Funnel stand - Rubber or plastic tubing - Clamp or spring clip - Cheesecloth or dental napkin - Thin stick or metal rod - Strainer - Microscope - Test tube | - Pasteur pipette - Small petri dishes - Scissors - Disposable paper towels - Spoon or spatula - Rubber band or length of string - Jug or flask - Microscope slides and coverslips - Iodine |
| --- | --- |

**Procedure**

- Take a funnel and fit a short piece of tubing to the stem. Close the tubing with a clamp or spring clip. Support the funnel on a single stand. If more than one sample requires processing at the same time use a plank in which holes have been made to support several funnels.
- If more than one sample requires processing at the same time use a plank in which holes have been made to support several funnels.
- Using a spoon or spatula weigh or measure approximately 5-10 grams of faecal material. Place the faecal material in the centre of the cheesecloth. Form a pouch containing the faecal material by holding the four corners of the cheesecloth together and moulding the cloth around the faecal material.
- Using a rubber band or length of string close the cheesecloth pouch. Push the stick or short metal rod under the rubber band or string so that the pouch can be suspended.
- Place the pouch containing the faecal material in the funnel.
- Trim off the excess cheesecloth.
- Fill the funnel with lukewarm water.
- Make sure the faecal material is covered.
- Leave the apparatus to stand for 24 hours.
- Draw off a few millilitres of fluid from the stem of the funnel into a test tube.
- Then either:
  - leave to sediment for at least 30 minutes.
  - Or if a centrifuge is available, the fluid can be drawn into a centrifuge tube and spun at 1000 rpm for 2 minutes.
- Check sedimented sample in a petri dish for the presence of larvae.
- This may be all that is required to diagnose the presence of nematode parasites but often more detailed examination is required.
- This is because other parasitic or free-living nematode life-cycle stages may be present if the [faecal sample](http://www.rvc.ac.uk/review/parasitology/Faeces/Purpose.htm) was not fresh when processed or if it was collected from the ground.
- Use a Pasteur pipette to transfer a small droplet of the sedimented fluid from the petri dish to a microscope slide.
- Add drop of iodine to fix the larvae and gently place a coverslip over the drop.
- Any free-living nematodes will stain dark brown very quickly while the larvae of parasitic species will only stain very slowly as the larval sheath protects the body.
- Examine under compound microscope at 10 x 10 magnification.
- Free-living nematodes stain deeply brown in iodine and can be distinguished by the presence of a double bulbed (rhabditiform) oesophagus (see figure).


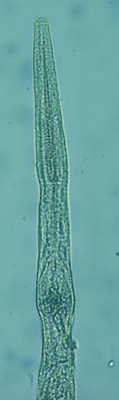

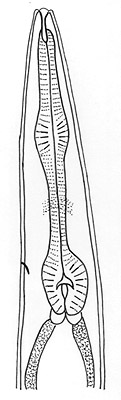


(Source: www.rvc.ac.uk/review/parasitology/baermann/examination.htm

**Faecal egg counts (eggs per gram epg) - Mc Master counting technique**

The McMaster counting technique is a quantitative technique to determine the number of eggs present per gram of faeces (e.p.g.). A flotation fluid is used to separate eggs from faecal material in a counting chamber (McMaster) with two compartments. The technique described below will detect 50 or more e.p.g. of faeces.

This technique can be used to provide a quantitative estimate of egg output for nematodes, cestodes and coccidia. Its use to quantify levels of infection is limited by the factors governing egg excretion.

**Equipment**

- Beakers or plastic containers
- Balance
- A tea strainer or cheesecloth
- Measuring cylinder
- Stirring device (fork, tongue depressor)
- Pasteur pipettes and (rubber) teats
- Flotation fluid (see the Appendix to this handbook for formulation)
- McMaster counting chamber*
- Microscope

**Procedure**

- Weigh 4 g of faeces and place into Container 1
- (Add 56 ml of flotation fluid
- (Mix (stir) the contents thoroughly with a stirring device (fork, tongue blade)
- Filter the faecal suspension through a tea strainer or a double-layer of cheesecloth into Container 2
- While stirring the filtrate in Container 2, take a sub-sample with a Pasteur pipette
- Fill both sides of the McMaster counting chamber with the sub-sample
- Allow the counting chamber to stand for 5 minutes (this is important)
- Examine the sub-sample of the filtrate under a microscope at 10 x 10 magnification.
- Count all eggs and coccidia oocytes within the engraved area of both chambers.
- The number of eggs per gram of faeces can be calculated as follows: Add the egg counts of the two chambers together.
- Multiply the total by 50. This gives the e.p.g. of faeces. (Example: 12 eggs seen in chamber 1 and 15 eggs seen in chamber 2 = (12 + 15) x 50 = 1350 e.p.g.)
- In the event that the McMaster is negative (no eggs seen), the filtrate in Container 2 can be used for the simple flotation method (section 3.2.2), steps f, g and h.

WARNING: In case of a time delay between processing the sample and reading the count, egg numbers may decline dramatically. Also, eggs may change their appearance, becoming crenated and "ghost-like". It is therefore advisable to prepare only a few samples at a time. These changes can be prevented by keeping prepared samples in the refrigerator after mixing. Using the salt-sugar solution as flotation fluid also reduces the morphological changes.

**Guideline to the interpretation of faecal egg counts in young animals**

| **Parasite** | | **Degree of infection (eggs per gram of faeces)** | | |
| --- | --- | --- | --- | --- |
|  |  | **Light** | **Moderate** | **Heavy** |
| CATTLE | | | | |
|  | Mixed infection | 50-200 | 200-800 | 800+ |
|  | Pure *Haemonchus* infection | 200 | 200-600 | 600+ |
|  | Pure Trichostrongylus infection | 50-100 | 100-400 | 400+ |
|  | Pure *Cooperia* infection | 200-300 | 300-2500 | 2500+ |
| SHEEP | | | | |
|  | Mixed infection | 50-800 | 800-1200 | 1200+ |
|  | Mixed infection with *Haemonchus* absent | 300-800 | 800-1000 | 1000+ |
|  | Pure *Haemonchus* | 100-2000 | 2000-7000 | 7000+ |
|  | Pure *Trichostrongylus* | 100-500 | 500-2000 | 2000+ |
|  | Pure *Nematodirus* | 50-100 | 100-600 | 600+ |
|  | Pure *Oesophagostomum* | 100-800 | 800-1600 | 1600+ |

Appendix D Working Instruction: collection of helminths (Necropsy at abattoir)

**COLLECTION OF HELMINTHS**

**Objectives**

1. To determine the presence or prevalence of *Haemonchus* and/or *Fasciola* in cattle

2. To determine the presence or prevalence of other nematodes

**Helminth recovery**

**Visual**

**Procedure** (This procedure takes more time but is more accurate) **(10 minutes)**

**Equipment**

1) Heavy-duty plastic bags

2) A ball of strong string or twine

3) 1 Sharp-pointed scissors

4) 1 Blunt-pointed scissors

5) 1 Fine-tipped forceps

***Haemonchus***

At slaughter separate the abomasum from the gastrointestinal tract by double ligatures tied at either end. Separate the liver from the other organs. Remove the abomasum from the rest of the gastrointestinal tract by cutting between the ligatures and place it in a large tray. (If it is not going to be processed immediately it can be placed in a heavy-duty plastic bag for the time being). Cut it open along its length using the blunt-pointed scissors. Carefully empty the contents of the abomasum into the tray and examine the abomasal mucosa and the upturned surface of the abomasal ingesta. If *Haemonchus* species are present the large females with red and white descending circles of colour, similar to a barber’s pole, are usually easily visible on the mucosa. They may also be present on the upturned surface of the abomasal ingesta but are less easily seen. The males are shorter and pink. Males and females can be collected using the fine-tipped forceps and identified under a stereoscopic microscope.

Adult hookworms (*Bunostomum* spp.) can be detected in the small intestine in the same way and adult *Oesophagostomum* can be detected in the large intestine in the same way.

***Fasciola***

Enlargement of the bile ducts and fibrosis surrounding the ducts are indications of fascioliosis. The bile ducts can be cut open along their lengths using the sharp-pointed scissors and the flukes exposed if they are present. Collect the flukes using the fine-tipped forceps and identify them under a stereoscopic microscope.

**Echinococcus** sp.

Incisions should be made in the liver and examined for the presence of hydatid cysts.

**Appendix E**  Working Instruction: Blood specimen collection and processing (smallholder farms)

**Cross-sectional survey**

Blood samples (approximately 125 μl) will be drawn from the middle ear vein using a capillary tube. To do this, cattle will be casted and immobilized. The middle ear vein is pricked with a blood lancet and blood is collected with an EDTA coated glass capillary. The blood sample is then applied onto a Whatman FTA card, air-dried and labeled appropriately and packed in foil pouches prior to DNA analysis.

**Isolation of blood parasites**

For live blood parasites, peripheral blood is aseptically punctured from the cattle’s tail vein into 5 ml glass vacutainers containing sodium heparin (tubes should be filled almost completely to reduce mortality of the parasites). These vacutainers can be stored on ice for several days until they processed in the laboratory.

Appendix F Packaging and transportation instructions

(2 pages including cover page)

**Regulations for UN3373 (**Packaging Requirements for Biological and Infectious Substances)(source: www.un3373.com/info/regulations)

Typically, air transport regulations are the most restrictive and the packaging specifications are the most rigorous. Shippers who comply with the air transport regulations also will meet the requirements of other transport modes. While there are some differences between the regulations for transport by air and by other modes (truck, rail, etc.), the vast majority of diagnostic specimens in the United States are packaged to comply with air transport requirements. Here, we have summarized the regulations of both the Department of Transportation (DOT) and the International Air Transport Association (IATA) into a single set of requirements. In cases where there are differences in meaning, we have used the more restrictive language.

If the hazardous materials you ship are UN 3373 “Biological substance, Category B,” “Diagnostic specimens”, “Clinical specimens,” or you ship medical or clinical waste, biological products, genetically modified micro-organisms, or other kinds of “infectious substances,” you should receive “General Awareness” hazardous materials training and “Infectious Substances” training. Those who prepare and ship specimens fitting the criteria for UN 3373 are required to know the requirements for proper transport.

What types of specimens meet the criteria of UN 3373? “Diagnostic” or “clinical specimens”, or “Biological substance, Category B” are substances which are known or are reasonably expected to contain pathogens and which are shipped for diagnostic or investigational purposes. Pathogens are defined as micro-organisms and other agents which can cause disease in humans or animals. Such pathogens are divided in two categories – A and B.

Category A

The pathogens listed in Category A present the greatest hazards to individuals and communities; and must be assigned and transported in compliance with 49 CFR, Part 173.196 or IATA Packing Instruction 602. They must be identified as either “UN 2814 Infectious substance, affecting humans” or “UN 2900 Infectious substance, affecting animals.” If exposure occurs, an infectious substance is capable of causing permanent disability, life-threatening or fatal disease to humans or animals.

Category B

Human or animal specimens which do not contain pathogens in Category A fall into Category B; and must be assigned to UN 3373 “Biological substance, Category B.” All UN 3373 substances must be transported in compliance with 49 CFR, Part 173.199 or IATA Packing Instruction 650.

Transport Packaging for UN 3373 Substances
Any packaging for biological substances must include three components:

• A primary receptacle: the tube, vial or other container typically made of glass or rigid plastic (including the stopper, cap or other closure elements) that is in direct contact with the specimen.
• A secondary packaging (including cushioning and other materials) that fully encapsulates the primary receptacle.

• An outer packaging for shipping or transit.Components must meet specific requirements, including being capable of passing specific test procedures based on receptacle or packaging type. In addition, compliance with the regulations is based, in part, on overall performance; so there can be no substitutions of a component from one manufacturer with a similar – but untested – component from another manufacturer.

Component Requirements:

• Under normal conditions of transport, primary receptacles must not break or leak their contents into secondary packagings.

• Multiple primary receptacles in the same secondary packaging must be separated to prevent contact between them.

• The performance of cushioning materials and the outer packaging must not be compromised due to any leakage from primary receptacles.

• For liquids, absorbent material sufficient to absorb the entire contents of all primary receptacles must be placed between the primary receptacles and the secondary packaging.

• One external surface of the outer packaging clearly must show the text “BIOLOGICAL SUBSTANCE, CATEGORY B.” Adjacent to this, inside a diamond mark whose lines are at least 2 mm thick, must appear the text “UN 3373” in characters at least 6 mm high.
Requirements for use of Ice, Dry Ice (carbon dioxide, solid), and Liquid Nitrogen as refrigerants for diagnostic or clinical specimens in transport:

• The primary receptacle(s) and secondary packaging must maintain their integrity and performance at the temperatures of the refrigerant used as well as at the temperatures and pressures that could result if refrigeration were lost.

• If ice or dry ice is used, it must be placed outside the secondary packaging, inside the outer packaging, or in an overpack. Also, the secondary packaging must be supported internally so that it maintains its original position while the ice melts or dry ice sublimates.
• If ice is used, the outer packaging must be leakproof and its performance must not be compromised by leakage of water.

• If dry ice is used, the packaging must allow for escape of carbon dioxide gas. In addition, the outer packaging must be marked with the text “Dry Ice” or “Carbon dioxide, solid” and “UN 1845” and the net quantity, in kilograms, of dry ice. Finally, these markings must be accompanied by the Class 9 label for Miscellaneous Dangerous Goods.

• When dry ice or liquid nitrogen is used, all other applicable requirements of the hazardous materials regulations must be met.

**Appendix G** Data Capture forms (DCFs)

| **DCF 1** | **ANIMAL DETAILS AND INCLUSION** |
| --- | --- |

Time = 24 hour clock Date format dd/mmm/yyyy

**Locality:**

| **Date**: | _____ / _________ / ______  dd / mmm / yyyy | | | | **Village name:** |  |
| --- | --- | --- | --- | --- | --- | --- |
| **Visit #:** | 🗆Visit 1 | 🗆Visit 2 | 🗆Visit 3 | 🗆Visit 4 | **Department or district** |  |
| **Owner name:** |  | | | | **Country:** |  |
| **GPS** | Latitude: Longitude: | | | | | |

**Animal identification:**

| **Animal ID:** |  | - |  | - |  | - |  |
| --- | --- | --- | --- | --- | --- | --- | --- |
|  | Country code^1^ |  | Dept/District code^2^ |  | Farm number |  | 3 digit animal number |

*^1^First two letters of country name; ^2^First letter of department or district name (if the same, first two)*

**Inclusion criteria:**

| **Is the estimated age of the animal between 1 and 2 years:** | 🗆 Yes 🗆 No |
| --- | --- |
| **Has the animal not received treatment with a topical or systemic acaricide/insecticide/anthelmintic for the last 2 weeks** | 🗆 Yes 🗆 No |

*If all the above is answered YES, the animal may be included for sampling*

**Animal details:**

| **Breed type:** |  | | | | | |
| --- | --- | --- | --- | --- | --- | --- |
| **Sex:** | 🗆 Male 🗆 Female | **Body weight:** | KG | | **Body condition score:** |  |
| **Pregnant or lactating?** | | 🗆 Yes 🗆 No 🗆 Not applicable | | | | |
| **Husbandry** | 🗆 Communal 🗆 Small trans humance 🗆 Large trans humance 🗆 Other | | | | | |
| **Has the animal been treated with any anti-parasitic drugs?** | | | | 🗆 Yes 🗆 No | | |
| **If YES, approximate time from last treatment:** | | | | weeks | | |
| **Name of product:** | | | | 🗆 Unknown | | |

| **Form completed by:** | Initial: Date: |
| --- | --- |

| **DCF 2** | **TICK IDENTIFICATION** |
| --- | --- |

Time = 24 hour clock Date format dd/mmm/yyyy

| **Sample ID:** | **BN - - -** | **Visit#:** | Visit 1 | Visit 2 | Visit 3 | Visit 4 |
| --- | --- | --- | --- | --- | --- | --- |

**Tick identification:**

| **Species^1^** | **Stage** | **Counts^2^** | **Number** |
| --- | --- | --- | --- |
|  |  |  |  |
|  |  |  |  |
|  |  |  |  |
|  |  |  |  |
|  |  |  |  |
|  |  |  |  |
|  |  |  |  |
|  |  |  |  |
|  |  |  |  |
|  |  |  |  |
|  |  |  |  |
|  |  |  |  |
|  |  |  |  |
|  |  |  |  |
|  |  |  |  |
|  |  |  |  |
|  |  |  |  |
|  |  |  |  |
|  |  |  |  |
|  |  |  |  |
|  |  |  |  |
|  |  |  |  |
|  |  |  |  |
|  |  |  |  |

^1^ If the species is unable to be identified write “sp.”

^2^ Put a mark for each tick identified

| **Species^1^** | **Stage** | **Counts^2^** | **Number** |
| --- | --- | --- | --- |
|  |  |  |  |
|  |  |  |  |
|  |  |  |  |
|  |  |  |  |
|  |  |  |  |
|  |  |  |  |
|  |  |  |  |
|  |  |  |  |
|  |  |  |  |
|  |  |  |  |
|  |  |  |  |
|  |  |  |  |
|  |  |  |  |
|  |  |  |  |
|  |  |  |  |
|  |  |  |  |
|  |  |  |  |
|  |  |  |  |
|  |  |  |  |
|  |  |  |  |
|  |  |  |  |
|  |  |  |  |
|  |  |  |  |
|  |  |  |  |
|  |  |  |  |
|  |  |  |  |
|  |  |  |  |

^1^ If the species is unable to be identified write “sp.”

^2^ Put a mark for each tick identified

| Ticks identified by: | Initial: date: |
| --- | --- |
| Recorded by: | Initial: date: |

| **DCF 3** | **HELMINTH EGGS IDENTIFICATION: SEDIMENTATION** |
| --- | --- |

Time = 24 hour clock Date format dd/mmm/yyyy

| **Sample ID:** | **BN - - -** | **Visit#:** | Visit 1 | Visit 2 | Visit 3 | Visit 4 |
| --- | --- | --- | --- | --- | --- | --- |

**Sedimentation technique:**

| **Species^1^** | **Counts^2^** | **Number** |
| --- | --- | --- |
|  |  |  |
|  |  |  |
|  |  |  |
|  |  |  |
|  |  |  |
|  |  |  |
|  |  |  |
|  |  |  |
|  |  |  |
|  |  |  |
|  |  |  |
|  |  |  |
|  |  |  |
|  |  |  |
|  |  |  |
|  |  |  |
|  |  |  |
|  |  |  |
|  |  |  |
|  |  |  |
|  |  |  |

^1^ If the species is unable to be identified write “sp.”

^2^ Put a mark for each tick identified

| Eggs identified by: | Initial: date: |
| --- | --- |
| Recorded by: | Initial: date: |

| **DCF 4** | **HELMINTH EGGS IDENTIFICATION: LARVAL CULTURE** |
| --- | --- |

Time = 24 hour clock Date format dd/mmm/yyyy

| **Sample ID:** | **BN - - -** | **Visit#:** | Visit 1 | Visit 2 | Visit 3 | Visit 4 |
| --- | --- | --- | --- | --- | --- | --- |

**Larval culture:**

| **Species^1^** | **Counts^2^** | **Number** |
| --- | --- | --- |
|  |  |  |
|  |  |  |
|  |  |  |
|  |  |  |
|  |  |  |
|  |  |  |
|  |  |  |
|  |  |  |
|  |  |  |
|  |  |  |
|  |  |  |
|  |  |  |
|  |  |  |
|  |  |  |
|  |  |  |
|  |  |  |
|  |  |  |
|  |  |  |
|  |  |  |
|  |  |  |

^1^ The species is difficult to determine but specify as good as possible the larva found

^2^ Put a mark for each larva identified

| Larvae identified by: | Initial: date: |
| --- | --- |
| Recorded by: | Initial: date: |

| **DCF 5** | **HELMINTH EGGS IDENTIFICATION: FLOATATION** |
| --- | --- |

Time = 24 hour clock Date format dd/mmm/yyyy

| **Sample ID:** | **BN - - -** | **Visit#:** | Visit 1 | Visit 2 | Visit 3 | Visit 4 |
| --- | --- | --- | --- | --- | --- | --- |

**Floatation technique:**

| **Species^1^** | **Counts^2^** | **Number** |
| --- | --- | --- |
|  |  |  |
|  |  |  |
|  |  |  |
|  |  |  |
|  |  |  |
|  |  |  |
|  |  |  |
|  |  |  |
|  |  |  |
|  |  |  |
|  |  |  |
|  |  |  |
|  |  |  |
|  |  |  |
|  |  |  |
|  |  |  |
|  |  |  |
|  |  |  |
|  |  |  |

^1^ The species is difficult to determine but specify as good as possible the eggs found: genus or family

^2^ Put a mark for each egg identified

| Eggs identified by: | Initial: date: |
| --- | --- |
| Recorded by: | Initial: date: |

| **DCF 6** | **HELMINTH EGGS IDENTIFICATION: FAECAL EGG COUNT** |
| --- | --- |

Time = 24 hour clock Date format dd/mmm/yyyy

| **Sample ID:** | **BN - - -** | **Visit#:** | Visit 1 | Visit 2 | Visit 3 | Visit 4 |
| --- | --- | --- | --- | --- | --- | --- |

**Faecal egg count (eggs per gram) - Mc Master counting technique:**

| **Species^1^** | **Egg count chamber 1** | **Egg count chamber 2** | **Total eggs** | **Eggs/gram^2^** |
| --- | --- | --- | --- | --- |
|  |  |  |  |  |
|  |  |  |  |  |
|  |  |  |  |  |
|  |  |  |  |  |
|  |  |  |  |  |
|  |  |  |  |  |
|  |  |  |  |  |
|  |  |  |  |  |
|  |  |  |  |  |
|  |  |  |  |  |
|  |  |  |  |  |
|  |  |  |  |  |
|  |  |  |  |  |
|  |  |  |  |  |
|  |  |  |  |  |
|  |  |  |  |  |
|  |  |  |  |  |
|  |  |  |  |  |
|  |  |  |  |  |

^1^ The species is difficult to determine but specify as good as possible the larva found

^2^ Eggs per gram can be calculated by taking the total number of eggs x 50. Follow the WI for the exact amount of faeces to be used and procedure to identify eggs as described in the WI

| Eggs identified by: | Initial: date: |
| --- | --- |
| Recorded by: | Initial: date: |

| **DCF 7** | **HELMINTH IDENTIFICATION: ADULT WORMS OR CYSTS** |
| --- | --- |

Time = 24 hour clock Date format dd/mmm/yyyy

| **Sample ID:** | **BN - - -** | **Visit#:** | Visit 1 | Visit 2 | Visit 3 | Visit 4 |
| --- | --- | --- | --- | --- | --- | --- |

**Abomasum:**

| **Species** | **Counts^1^** | **Total** |
| --- | --- | --- |
|  |  |  |
|  |  |  |
|  |  |  |
|  |  |  |

^1^ Put a mark for each worm identified

**Liver**:

| **Species** | **Counts^1^** | **Total** |
| --- | --- | --- |
|  |  |  |
|  |  |  |
|  |  |  |
|  |  |  |

^1^ Put a mark for each worm or hydatic cyst identified

**Small intestine:**

| **Species** | **Counts^1^** | **Total** |
| --- | --- | --- |
|  |  |  |
|  |  |  |
|  |  |  |
|  |  |  |

^1^ Put a mark for each larva identified

**Large intestine:**

| **Species** | **Counts^1^** | **Total** |
| --- | --- | --- |
|  |  |  |
|  |  |  |
|  |  |  |
|  |  |  |

^1^ Put a mark for each worm identified

**Bile duct:**

| **Species** | **Counts^1^** | **Total** |
| --- | --- | --- |
|  |  |  |
|  |  |  |
|  |  |  |
|  |  |  |

^1^ Put a mark for each worm identified

| Worms identified by: | Initial: date: |
| --- | --- |
| Recorded by: | Initial: date: |

| **DCF 8** | **ABATTOIR SAMPLING** |
| --- | --- |

Time = 24 hour clock Date format dd/mmm/yyyy

**Locality:**

| **Date**: | _____ / _________ / ______  dd / mmm / yyyy | **Village name:** |  |
| --- | --- | --- | --- |
|  | | **Department:** |  |
| **Abattoir name:** |  | **Country:** |  |
| **GPS** | Latitude: Longitude: | | |

**Sample identification:**

| **Sample ID:** | BN | - |  | - |  | - |  | - |  |
| --- | --- | --- | --- | --- | --- | --- | --- | --- | --- |
|  | Country code^1^ |  | Department code^2^ |  | Abattoir number |  | Animal number |  | organ^3^ |

*^1^BN = Benin; ^2^Donga: D, Zou: Z*; ^3^Abomasum: A, small/large intestine: I, liver: L

**Questionnaire:**

| What is the reason for slaughtering this animal? | 🗆 Age 🗆 Disease 🗆 Ceremony 🗆 Cash 🗆 Injury  🗆 Other: ………………………………… |
| --- | --- |
| How many animals do you slaughter a year? | 🗆 1 🗆 2 - 5 🗆 >5 |
| How many animals do you own? | 🗆 1 - 10 🗆 11 - 20 🗆 >20 |

**Samples collected:**

| Sample collected from abomasum? | 🗆 Yes 🗆 No | BN - - - - A |
| --- | --- | --- |
| Sample collected from small or large intestines? | 🗆 Yes 🗆 No | BN - - - - I |
| Sample collected from liver? | 🗆 Yes 🗆 No | BN - - - - L |

| **Form completed by:** | Initial: Date: |
| --- | --- |

| **DCF 9** | **Sample collection and half body tick counts (page 1 of 2)** |
| --- | --- |

Time = 24 hour clock Date format dd/mmm/yyyy

| **Animal ID:** | **BN - - -** | **Visit#:** | Visit 1 | Visit 2 | Visit 3 | Visit 4 |
| --- | --- | --- | --- | --- | --- | --- |

**Sample collection:**

|  |  | **Sample ID^1^** |
| --- | --- | --- |
| **Blood sample collected on Whatman® FTA® card?** | 🗆 **Yes** 🗆 **No** | **BN - - - - B** |
| **Faecal sample collected?** | 🗆 **Yes** 🗆 **No** | **BN - - - - F** |

*1 Transcribe animal ID assigned on DCF 1 and use this ID for sample identification*

| Samples taken by: | Initial: date: |
| --- | --- |
| Recorded by: | Initial: date: |

**Half body tick count, fill in number of ticks counted in each area:**

|  |  |  | 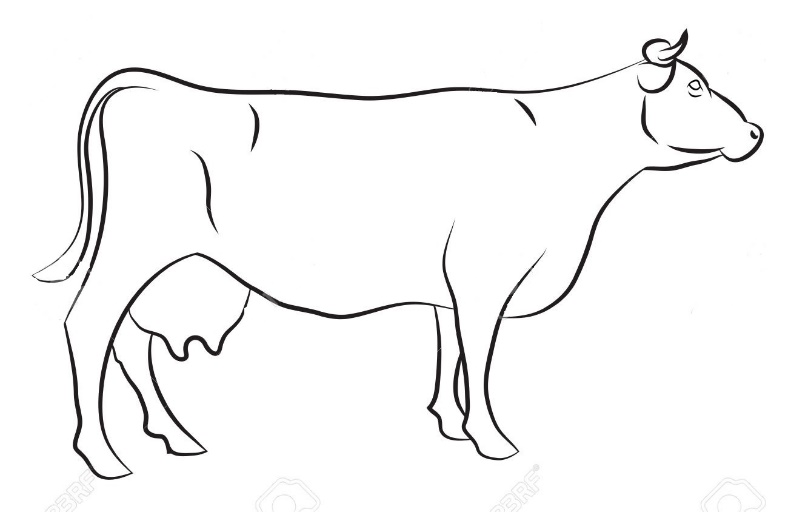  **3**  **1**  **4**  **2**  **5** |
| --- | --- | --- | --- |
|  |  |  |  |
|  |  |  |  |
| 1. Inner and outer fore-legs, hind legs and abdomen | |  |  |
| 2. Tail and anal area | |  |  |
| 3. Head and neck , | |  |  |
| 4. Lateral area and dorsal area shoulders to tail base | |  |  |
| 5. Ears | |  |  |

| **Tick type** | **Number of adult ticks counted per body area** | | | | |
| --- | --- | --- | --- | --- | --- |
|  | **1** | **2** | **3** | **4** | **5** |
| ***Hyalomma*** |  |  |  |  |  |
| ***Amblyomma*** |  |  |  |  |  |
| ***Rhipicephalus*** |  |  |  |  |  |
| ***Boophilus*** |  |  |  |  |  |
| **Genus not identifiable** |  |  |  |  |  |

| Ticks counted by: | Initial: date: |
| --- | --- |
| Recorded by: | Initial: date: |

| **DCF 9** | **Sample collection and half body tick counts (page 2 of 2)** |
| --- | --- |

Time = 24 hour clock Date format dd/mmm/yyyy

| **Animal ID:** | **BN - - -** | **Visit#:** | Visit 1 | Visit 2 | Visit 3 | Visit 4 |
| --- | --- | --- | --- | --- | --- | --- |

| Ticks counted by: | Initial: date: |
| --- | --- |
| Recorded by: | Initial: date: |

| **DCF 10** | **QUESTIONNAIRE: DEMOGRAPHIC AND SOCIO-ECONOMIC PARAMETERS** |
| --- | --- |

Time = 24 hour clock Date format dd/mmm/yyyy

**Locality:**

| **Date**: | _____ / _________ / ______  dd / mmm / yyyy | | | | **Village name:** |  |
| --- | --- | --- | --- | --- | --- | --- |
| **Visit #:** | 🗆Visit 1 | 🗆Visit 2 | 🗆Visit 3 | 🗆Visit 4 | **Deptm/district:** |  |
| **Owner name:** |  | | | | **Country:** |  |
| **GPS** | Latitude: Longitude: | | | | | |

**Owner identification:**

| **Owner ID:** |  | - |  | - |  |
| --- | --- | --- | --- | --- | --- |
|  | Country code^1^ |  | Deptm/District code^2^ |  | Farm number |

*^1^First two letters of country name; ^2^First letter of department or district name (if the same, first two)*

**Inclusion criteria:**

| **Are there any animals enrolled in the study from this farm** | 🗆 Yes 🗆 No |
| --- | --- |

*If the above is answered YES, the farmer may be included in the questionnaire*

**Land assets:**

| Parc* ID | Parcel Description / Name | Size of parcel | | Unit of land (Code) | Tenure system  (Code) | If parcel **is *owned,*** who owns (Code**)** |
| --- | --- | --- | --- | --- | --- | --- |
| 1 |  |  | |  |  |  |
| 2 |  |  | |  |  |  |
| 3 |  |  | |  |  |  |
| **UNIT OF LAND**  1= acre  2= ha  3= sqm2  4= other, specify conversion in metric system | | | **TENURE SYSTEM**  1= Title deed  2= Owned but not titled  3= public land  4= Rented‐in/ sharecropped  5=Other (specify) | | **If owned, name on title/certificate:**  1= Male  2= Female  3= Joint  4=Other relative  5= Other | |

| **DCF 11** | **QUESTIONNAIRE: ANIMAL HEALTH INTERVENTIONS** |
| --- | --- |

Time = 24 hour clock Date format dd/mmm/yyyy

**Locality:**

| **Date**: | _____ / _________ / ______  dd / mmm / yyyy | | | | **Village name:** |  |
| --- | --- | --- | --- | --- | --- | --- |
| **Visit #:** | 🗆Visit 1 | 🗆Visit 2 | 🗆Visit 3 | 🗆Visit 4 | **Deptm/district:** |  |
| **Owner name:** |  | | | | **Country:** |  |
| **GPS** | Latitude: Longitude: | | | | | |

**Owner identification:**

| **Owner ID:** |  | - |  | - |  |
| --- | --- | --- | --- | --- | --- |
|  | Country code^1^ |  | Deptm/district code^2^ |  | Farm number |

*^1^First two letters of country name; ^2^First letter of department or district name (if the same, first two)*

**Inclusion criteria:**

| **Are there any animals enrolled in the study from this farm** | 🗆 Yes 🗆 No |
| --- | --- |

*If the above is answered YES, the farmer may be included in the questionnaire*

**Use of drugs**

Since this time last year, have you purchased any veterinary drugs to treat sick animals?

□ Yes □ No □ Don‘t know

**If no**, why did you not buy veterinary drugs to treat your sick animals *(tick one of more appropriate box(es))*

1. Because I could not afford to pay for the veterinary drugs I needed □
2. Because none of my animals got sick during the year □
3. Because I don’t think they are effective □
4. Because I don’t think it was necessary □
5. Because you cannot buy veterinary drugs in this area □
6. Because I do not know which veterinary drugs to buy □
7. Other reasons □ (specify)……………………………………………………………………………………………………………………………………………………………………………………………………

**If yes**, who do you normally buy your veterinary drugs from *(tick one of more appropriate box(es))*

1. From the government veterinary office in the nearest main town □
2. From the local government Veterinary Assistant (VA) □
3. From a private trader □
4. From private veterinary practitioners □
5. From community animal health workers (CAHW) □
6. From other farmers □
7. From cooperatives/ farmer organisations □
8. From an NGO □
9. From another source □

(specify)…………………………………………………………………………………………………………………………………………………………………………………………………………….

**If yes**, who administered the veterinary drugs *(tick one of more appropriate box(es))*

1. Myself/ my neighbour □
2. The local government Veterinary Assistant (VA) □
3. A Privat veterinary practitioner □
4. A community animal health worker (CAHW) □
5. Other (specify)………………………… □

**Preventive measures**

Are there any preventive treatments/vaccinations available for animals? □ Yes □ No □ Don’t know

Have any of your animals been vaccinated in the *last* *12 months*? □ Yes □ No □ Don’t know

For every *animal* *species* a selection of *diseases or vaccines* are given, for every disease or vaccine report whether you *applied it or not* and give the *reasons* for using or not using (more answers can be given), indicate *who* administered the vaccine and *where* you got it from (source of vaccine), also report the *frequency* of use and whether you are *satisfied* with the results of that vaccine.

| **Vaccine**  List to be complete after advice from vet services | **Y/N** | **Reason for (not) using**  *(use number from list below)* | **Who vaccinated**  *(use number from list below)* | | **Source of vaccine**  *(use number from list below)* | **Frequency of use**  *(use number from list below)* | **Satisfaction**  *(use number from list below)* | |
| --- | --- | --- | --- | --- | --- | --- | --- | --- |
| **Cattle** |  |  |  | |  |  | |  |
| FMD |  |  |  | |  |  | |  |
| LSD |  |  |  | |  |  | |  |
| BQ |  |  |  | |  |  | |  |
| Anthrax |  |  |  | |  |  | |  |
| Brucella |  |  |  | |  |  | |  |
| ECF |  |  |  | |  |  | |  |
|  |  |  |  | |  |  | |  |
|  |  |  |  | |  |  | |  |
| **Goats** |  |  |  | |  |  | |  |
|  |  |  |  | |  |  | |  |
|  |  |  |  | |  |  | |  |
| **Poultry** |  |  |  | |  |  | |  |
| Newcastle disease |  |  |  | |  |  | |  |
|  |  |  |  | |  |  | |  |
| **Sheep** |  |  |  | |  |  | |  |
|  |  |  |  | |  |  | |  |
|  |  |  |  | |  |  | |  |
| **Dogs** |  |  |  | |  |  | |  |
| Rabies |  |  |  | |  |  | |  |
|  |  |  |  | |  |  | |  |
| **Reasons for using**  1= vaccination compulsory and free  2= vaccination results in fewer sick animals and mortality  3= vaccination is the most efficient disease control strategy  4= I can keep grade cattle after vaccinating  5= I can keep more cattle after vaccinating  6= Other (specify) | | | | **Reasons for not using**  1= I cannot afford the vaccine  2= the vaccine is not available  3= the vaccine not effective  4= the vaccination not necessary  5= the vaccination causes too many side-effects  6= the vaccination too cumbersome  7= other (specify) | | **Who vaccinated**  1= Myself/ neighbour  2= local government VA  3= private veterinarian  4= CAHW  5= NGO vet  6=other (specify) | | |

**If no**, why do you never use vaccines? *(tick one of more appropriate box(es))*

1. Because I could not afford to pay for vaccines □
2. Because none of my animals got sick during the year □
3. Because I don’t think the vaccines are effective □
4. Because I don’t think the vaccines are necessary □
5. Because you cannot buy vaccines in this area □
6. Because I do not know which vaccines to buy □
7. Other reasons □

(specify)…………………………………………………………………………………………………………………………………………………………………………………………..

**Ectoparasite control**

Do you control **ectoparasites** (ticks, lice, flies…) in animals? □ Yes □ No □ Don’t know

**If yes,** do you do it

1. routinely □
2. only when problems arise □

When *previous answer = option 2*, do you then use the treatment on

both sick and healthy animals □

only the animals that are sick □

**If yes,** specify the *animal species*, the *specific drug* (product name) used *or* the *traditional remedy* used, the *method* how it’s applied and the *frequency*

| **Species**  *(use number from list below)* | **Drug/ Traditional remedy used**  *(indicate product name or name of traditional remedy)* | **Method**  *(use number from list below)* | **Frequency**  *(use number from list below)* |
| --- | --- | --- | --- |
|  |  |  |  |
|  |  |  |  |
|  |  |  |  |
|  |  |  |  |

| **Species** | **Method** | **Frequency** |
| --- | --- | --- |
| 1= Cattle  2= Goats  3= Sheep  4= Pigs  5= Poultry  6= Donkeys/equines  7= Dogs | 1= Spraying of legs only  2= Spraying of whole body  3= Dipping  4= Pour-on  5= Manual removal  6= Injectables  7= Other | 1= once a week  2= twice a month  3= once a month  4= less frequent |

|  |
| --- |

**If no**, why do you not control ectoparasites *(tick one of more appropriate box(es))*

1. Because I cannot afford to pay for it □
2. Because ectoparasites do not cause problems in my animals □
3. Because I don’t believe it is effective □
4. Because I don’t think it is necessary □
5. Because products to control ectoparasites are not available in this area □
6. Because I do not know which product to use/ buy □
7. Other reasons □

(specify)……………………………………………………………………………………………

**Endoparasite control**

Do you control **intestinal parasites** in animals? □ Yes □ No □ Don’t know

**If yes,** do you do it

- - 1. routinely □
    2. only when problems arise □

When *previous answer = option 2* do you then use the treatment on

1. both sick and healthy animals □
2. only the animals that are sick □

**If yes,** specify the *animal species*, the *specific drug* used or the *traditional remedy* used, the *method* how it’s applied and the *frequency*

| **Species**  *(use number from list below)* | **Drug/ Traditional remedy used**  *(indicate product name or name of traditional remedy)* | **Method**  *(use number from list below)* | **Frequency**  *(use number from list below)* |
| --- | --- | --- | --- |
|  |  |  |  |
|  |  |  |  |
|  |  |  |  |
|  |  |  |  |

| **Species** | **Method** | **Fequency** |
| --- | --- | --- |
| 1= Cattle  2= Goats  3= Sheep  4= Pigs  5= Poultry  6= Donkeys/equines  7= Dogs | 1= Drench  2= Bolus  3= Feed  4= Injection  5= Pasta  6= Other  7= I don’t know | 1= once a week  2= twice a year  3= once a year  4= less frequent |

**If no**, why do you not control intestinal parasites *(tick one of more appropriate box(es))*

1. Because I cannot afford to pay for it □
2. Because intestinal parasites do not cause problems in my animals □
3. Because I don’t believe it is effective □
4. Because I don’t think it is necessary □
5. Because you cannot buy products to control intestinal parasites in this area □
6. Because I do not know which product to use/buy □
7. Other reasons □ (specify)……………………………………………………………………………………..

| **Form completed by:** | Initial: Date: |
| --- | --- |

**Farm and domestic assets:**

| Name of Asset | Total # owned | Relative/ average age  (number in this age group) | | |
| --- | --- | --- | --- | --- |
|  |  | <3 years | 3‐7 years | > 7 years |
| **Domestic** | | | | |
| Cooker/gas stove |  |  |  |  |
| Refrigerator |  |  |  |  |
| Radio |  |  |  |  |
| Television |  |  |  |  |
| DVD player |  |  |  |  |
| Mobile phone |  |  |  |  |
| Sofa set |  |  |  |  |
| Sewing machine |  |  |  |  |
| Mosquito nets |  |  |  |  |
| **Transport** | | | | |
| Car/truck |  |  |  |  |
| Motorcycle |  |  |  |  |
| Bicycle |  |  |  |  |
| Cart (animal drawn) |  |  |  |  |
| **Farm** | | | | |
| Hoes |  |  |  |  |
| Spades/shovel |  |  |  |  |
| Ploughs |  |  |  |  |
| Sprayer pump |  |  |  |  |
| Water pump |  |  |  |  |
| Other asset 1 |  |  |  |  |
| Other asset 2 |  |  |  |  |

**Housing:**

| Home ownership | Number of rooms | Floor material (Code) | Wall material  (code) | Roofing material (code) |
| --- | --- | --- | --- | --- |
|  |  |  |  |  |
|  |  | **FLOOR MATERIAL** | **WALL MATERIAL** | **ROOFING MATE** |
| 1= Owned  2= Rented  3= Borrowed  4= Other (specify) |  | 1= earth  2= cement  3= tiles  4= other, specify | 1= earth/mud  2= wood/bamboo/  iron sheets  3= cement/brick  4= other, specify | 1= grass  2= iron sheets/asbestos  3= tiles  4= other, specify |

**Livestock:**

| **Livestock species** | | **Number owned by household** |
| --- | --- | --- |
| **Cattle** | **Local** |  |
|  | **Cross/exotic** |  |
| **Sheep** | **Local** |  |
|  | **Cross/exotic** |  |
| **Goats** | **Local** |  |
|  | **Cross/exotic** |  |
| **Poultry** | **Local** |  |
|  | **Cross/exotic** |  |
| **Pigs** | **Local** |  |
|  | **Cross/exotic** |  |
| **Horses/donkeys** | |  |
| **Other, specify** | |  |
|  | |  |

| **Form completed by:** | Initial: Date: |
| --- | --- |

dddddddddddddddd
